# Molecular mechanism of GIRK2 channel gating modulated by cholesteryl hemisuccinate

**DOI:** 10.3389/fphys.2024.1486362

**Published:** 2024-10-18

**Authors:** Meng Cui, Yongcheng Lu, Xinyi Ma, Diomedes E. Logothetis

**Affiliations:** ^1^ Department of Pharmaceutical Sciences, School of Pharmacy, Bouvé College of Health Sciences, Northeastern University, Boston, MA, United States; ^2^ Center for Drug Discovery, Northeastern University, Boston, MA, United States; ^3^ Affiliate of Chemistry and Chemical Biology, Northeastern University, Boston, MA, United States; ^4^ Affiliate of Bioengineering, Northeastern University, Boston, MA, United States; ^5^ Affiliate of Roux Institute of Northeastern University, Portland, ME, United States

**Keywords:** GIRK channel, cholesterol, MD simulations, protein-ligand interactions, ion channel activation, conformational changes, interaction network analysis, electrostatic potential

## Abstract

Cholesterol, an essential lipid of cell membranes, regulates G protein-gated inwardly rectifying potassium (GIRK) channel activity. Previous studies have shown that cholesterol activates GIRK2 homotetrameric channels, which are expressed in dopaminergic neurons of the brain. Deletion of GIRK2 channels affects both GIRK2 homo- and heterotetrames and can lead to abnormal neuronal excitability, including conditions such as epilepsy and addiction. A 3.5 Å cryo-EM structure of GIRK2 in complex with CHS (cholesteryl hemisuccinate) and PIP_2_ (phosphatidylinositol 4,5-bisphosphate) has been solved. This structure provides the opportunity to study GIRK2 channel gating dynamics regulated by cholesterol using gating molecular dynamics (GMD) simulations. In the present study, we conducted microsecond-long GMD simulations on the GIRK2 channel in its APO, PIP_2_, and PIP_2_/CHS bound states, followed by systematic analysis to gain molecular insights into how CHS modulates GIRK2 channel gating. We found that CHS binding facilitates GIRK2 channel opening, with 43 K^+^ ion permeation events observed, compared to 0 and 2 K^+^ ion permeation events for GIRK2-APO and GIRK2/PIP_2_, respectively. Binding of CHS to the GIRK2 channel enhances PIP_2_ and channel interactions, which is consistent with previous experimental results. The negatively charged PIP_2_ alters the internal electrostatic potential field in the channel and lowers the negative free energy barrier for K^+^ ion permeation.

## Introduction

G protein-gated inwardly rectifying potassium (Kir3 or GIRK) channels, activated directly by the Gβγ subunits of heterotrimeric G proteins, mediate the inhibitory effects of various neurotransmitters in excitable cells, such as in the heart and the nervous system. Four GIRK channel family members (GIRK1, GIRK2, GIRK3, and GIRK4) have been identified. GIRK channels can be expressed either as homotetramers (GIRK2 or GIRK4) or as heterotetramers with the non-functional homomeric subunits (GIRK1 and GIRK3) (Cui et al., 2021). In the atria of the heart, GIRK1 and GIRK4 subunits form K_ACh_ channels, which can be activated by acetylcholine via muscarinic M2 receptors coupled to pertussis (PTX)-sensitive Gi/o proteins to regulate heart rate (Dascal, 1997; Ivanova-Nikolova et al., 1998). Loss of function mutations in humans have been described to cause Long QT (LQT) syndrome (Amadei et al., 1999), while gain of function mutations cause atrial fibrillation (AF) (Cui et al., 2021).

In the brain, dopaminergic neurons within the ventral tegmental area (VTA) express GIRK2 and GIRK3, producing GIRK2 homotetramers as well as GIRK2/GIRK3 heterotetramers (Jelacic et al., 2000; Cruz et al., 2004; Kotecki et al., 2015). GABAergic neurons within the VTA also express GIRK2 and GIRK3 along with GIRK1, producing the dominating GIRK1/GIRK2 heterotetramers (Cui et al., 2021). Multiple inhibitory neurotransmitters, including ACh, adenosine, dopamine, opioids, GABA, bind their respective Gi/o protein-coupled to activate GIRK channels using the G_βγ_ subunits of PTX-sensitive G proteins (Logothetis et al., 1987; Cui et al., 2021). Activation of GIRK channels allowing transitory potassium efflux, which lowers the resting membrane potential of neurons and reduces their excitability (Dascal, 1997). Deletion of GIRK channels in the brain cause abnormal neuronal excitability, including epilepsy and addiction (Zhao et al., 2020).

GIRK channels can be activated by various modulators, including G_βγ_ (Logothetis et al., 1987), Na^+^ ions (Sui et al., 1996; Ho and Murrell-Lagnado, 1999; Zhang et al., 1999), and alcohols (Kobayashi et al., 1999; Lewohl et al., 1999). It was shown that GIRK channel activation by these modulators or drugs requires the presence of PIP_2_ (Huang et al., 1998; Sui et al., 1998; Logothetis and Zhang, 1999; Petit-Jacques et al., 1999; Meng et al., 2012; Meng et al., 2016; Li et al., 2019; Gazgalis et al., 2022). It seems that these gating molecules activate GIRK channels by strengthening the interactions of the channel with PIP_2_ (Zhang et al., 1999).

GIRK channels can also be activated by cholesterol (Rosenhouse-Dantsker et al., 2010), an essential lipid that plays critical roles in maintaining the integrity and fluidity of cell membranes and serves as a precursor for the synthesis of vital substances such as steroid hormones, bile acids, and vitamin D (Zampelas and Magriplis, 2019; Rosenhouse-Dantsker et al., 2023). High levels of cholesterol in the brain have been implicated in neurodegenerative diseases such as Alzheimer’s and Parkinson’s (Mathiharan et al., 2021).

To better understand the molecular basis by which cholesterol activates GIRK channels, a cryo-EM structure of GIRK2 has been solved in complex with CHS (cholesteryl hemisuccinate, a cholesterol analog) and diC8-PIP_2_ (a short acyl chain soluble analog of PIP_2_). Two CHS binding sites were identified in the GIRK2 cryo-EM structures, both of which are close to the PIP_2_ binding site (Mathiharan et al., 2021). The cryo-EM structures illuminate the molecular architecture of the cholesterol binding site on GIRK2 channels and provide an opportunity to study GIRK2 channel gating dynamics regulated by cholesterol using gating molecular dynamics (GMD) simulations. In this study, we conducted microsecond-long GMD simulations on three systems: GIRK2-APO, GIRK2/PIP_2_, and GIRK2/PIP_2_/CHS. We have systematically analyzed the GMD trajectories to gain molecular insights into how PIP_2_ and CHS modulate GIRK2 channel gating.

## Materials and methods

### Molecular docking

There are two CHS binding sites (A and B) observed in the GIRK2 channel cryo-EM structure (PDBID: 6XEV) (Mathiharan et al., 2021). However, only CHS in site A was solved in the structure. The CHS was docked into the binding site B (based on the density map and binding site residues) in the GIRK2 channel using induced-fit-docking (IFD) simulations (Schrodinger, Inc.) (Sherman et al., 2006). Default parameters were used for IFD simulations. The residues within 5 Å of ligand poses were selected for side chain optimization by prime refinement. XP scores were used for ranking of the ligand poses, and the top 5 poses of the docked ligand were saved for visual inspection and selection. The pose of the docked ligand was selected by a comparison with a previously published pose which was consistent with cryo-EM density for the CHS binding site B (Mathiharan et al., 2021), and used it as the predicted pose for MD simulations.

### GMD simulations

Protonation states of the titratable residues in GIRK2 channel were calculated at pH = 7.4 via the use of the H++ server (http://biophysics.cs.vt.edu/) (Gordon et al., 2005). The GIRK2-APO, GIRK2/PIP_2_ or GIRK2/PIP_2_/CHS complexes were inserted into a simulated lipid bilayer composed of POPC:POPE:POPS:cholesterol (25:5:5:1) (Leal-Pinto et al., 1995) and a water box using the CHARMM-GUI Membrane Builder webserver (http://www.charmm-gui.org) (Jo et al., 2008). Potassium chloride (150 mM) as well as neutralizing counter ions were applied to the systems. Four sodium ions were added to the Na^+^ binding sites of GIRK2 (Whorton and MacKinnon, 2011). The total atom numbers are 214,784; 215,400; and 216,078 for the GIRK2-APO, GIR2/PIP_2_ and GIRK2/PIP_2_/CHS, respectively. The PMEMD.CUDA program of AMBER 20 was used to conduct MD simulations (Case et al., 2005). The Amber ff14SB, lipid17 and TIP3P force field were used for the channels, lipids, and water. The parameters of CHS and PIP_2_ were generated using the general AMBER force field by the Antechamber module of AmberTools 17 and using the partial charge determined via restrained electrostatic potential charge-fitting scheme by *ab initio* quantum chemistry at the HF/6–31G* level (Breneman and Wiberg, 1990). Coordinate files and system topology were established using the tleap module of Amber. The systems were energetically minimized by 500 steps (with position restraint of 500 kcal/mol/Å^2^) followed by 2,000 steps (without position restraint) using the steepest descent algorithm. Heat was then applied to the systems to drive the temperature from 0 to 303 K using Langevin dynamics with a collision frequency of 1 ps^-1^. The channel complexes were position-restrained using an initial constant force of 500 kcal/mol/Å^2^ during the heating process, subsequently diminished to 10 kcal/mol/Å^2^, allowing the lipid and water molecules free movement. Before the MD simulations, the systems underwent 5 ns equilibration. Then, a total of 1µs of MD simulations were conducted using hydrogen mass repartitioning and a time step of 4 fs. The coordinates were saved every 100 ps for analysis. The simulations were conducted in an isothermal and isobaric nature, with the pressure maintained using an isotropic position scaling algorithm with the pressure relaxation time fixed at 2 ps. An external voltage of 0.06 V/nm (approximately 200 mV across the membrane) was added to the systems from the extracellular to the intracellular side (Meng et al., 2012; Meng et al., 2016; Li et al., 2019; Cui et al., 2022). Long-range electrostatics were calculated by a particle mesh Ewald method with a 10 Å cut-off (Darden et al., 1993). We call these MD simulations Gating (GMD) as we can see gating and ion permeation in the course of the simulation. The results of the GMD simulations were analyzed using various tools and methods, including the built-in utility of the GROMACS program from Groningen University, Simulaid (Mezei, 2010), as well as in-house scripts.

### Binding free energy calculations

The MM-GBSA module, which is implemented in Amber18, was performed to calculate the binding free energy of PIP_2_ with the GIRK2 channel. The binding free energy can be decomposed into contributions from individual interacting residues of the channels, which consist of four energy terms: non-bonded electrostatic interaction, van der Waals energy in gas phase, polar, and non-polar solvation free energy. The polar component is calculated using a Generalized Born (GB) implicit solvation model. The non-polar component is calculated using a solvent accessible surface area model. 100 snapshots were extracted from the 200–1,000 ns MD simulation trajectory at an interval of 80 ns for the binding free energy calculations.

## Results

### CHS facilitates homomeric GIRK2 channel openings in the presence of PIP_2_

**FIGURE 1:**
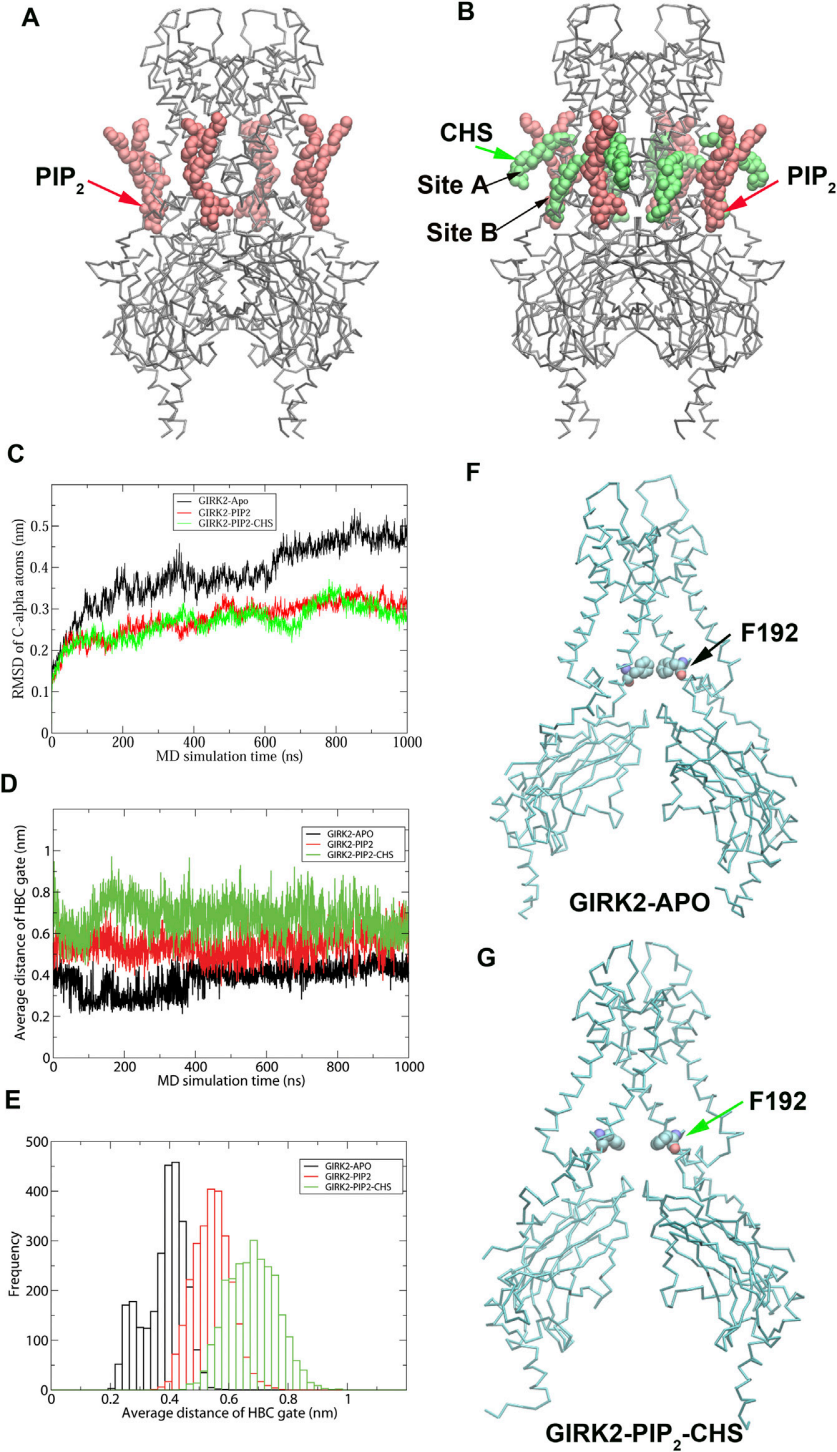
MD simulation results on GIRK2-APO, GIRK2-PIP_2_ and GIRK-PIP_2_-CHS systems **(A)**. GIRK2-PIP_2_: GIRK2 channel bound with 4 PIP_2_ molecules. **(B)**. GIRK2-PIP_2_-CHS: GIRK2 channel bound with 4 PIP_2_ and 8 CHS molecules. **(C)**. RMSD of Cα atoms of the GIRK2 channels for the three systems as function of simulation time (ns). **(D)**. HBC gate residues’ distances as function of simulation time (ns). **(E).** Histogram of **(D)**. **(F)**. Residue F192 of the HBC gate in GIRK2-APO (262 ns). **(G)**. Residue F192 of the HBC gate in GIRK2-PIP_2_-CHS (384 ns).

To understand CHS-induced conformational changes in GIRK2 channel, We performed 1 µs MD simulations on the GIRK2 (APO), GIRK2/PIP2, and GIRK2/PIP2/CHS systems, each including four bound Na^+^ ions. The complex structure of GIRK2/PIP2/CHS was obtained from the Protein Data Bank (PDBID: 6XEV). Four diC_8_ PIP_2_ molecules were replaced by the full length PIP_2_ (arachidonyl/stearyl). Since the site B bound CHS was missing in the cryo-EM structure, we predicted the binding pose of CHS in site B using molecular docking simulations based on the density maps and key residues suggested in the paper (Mathiharan et al., 2021). Supplementary Figure S1 shows the sequence of mouse GIRK2 channel marked with secondary structure regions and motifs. Figures 1A, B shows the initial structures of GIRK2/PIP_2_ and GIRK2/PIP_2_/CHS, respectively. Figure 1C shows the RMSD of Cα atoms of GIRK2 as function of simulation time. Changes in RMSD were minimal for the three systems following 200 ns of simulation. However, the RMSD for GIRK2-APO increased between 600 ns and 1µs simulations, which may indicate conformational changes from the initial structure in the absence of PIP_2_ and CHS. In contrast, the systems with PIP_2_ or PIP_2_/CHS showed relatively small changes in the RMSD. Figures 1D, E show the distances of the HBC gate changes as a function of simulation time, and gate distance distributions in these three systems, respectively. The results indicate that PIP_2_ or PIP_2_/CHS facilitates GIRK2 channel openings in comparison to the GIRK2-APO state. Figures 1F, G show the representative snapshots in GIRK2-APO and GIRK2/PIP_2_/CHS systems from MD simulations, respectively. Root mean square fluctuation (RMSF) of the Cα atoms of GIRK2-APO, and GIRK2-PIP_2_-CHS are given in Supplementary Figure S2. The results show that among other difference in these two systems, the S-HLX (SH) in the GIRK2-PIP_2_-CHS system is more stable compared to the GIRK2-APO system, while the SF region becomes more flexible in the GIRK2-PIP_2_-CHS system.

**FIGURE 2:**
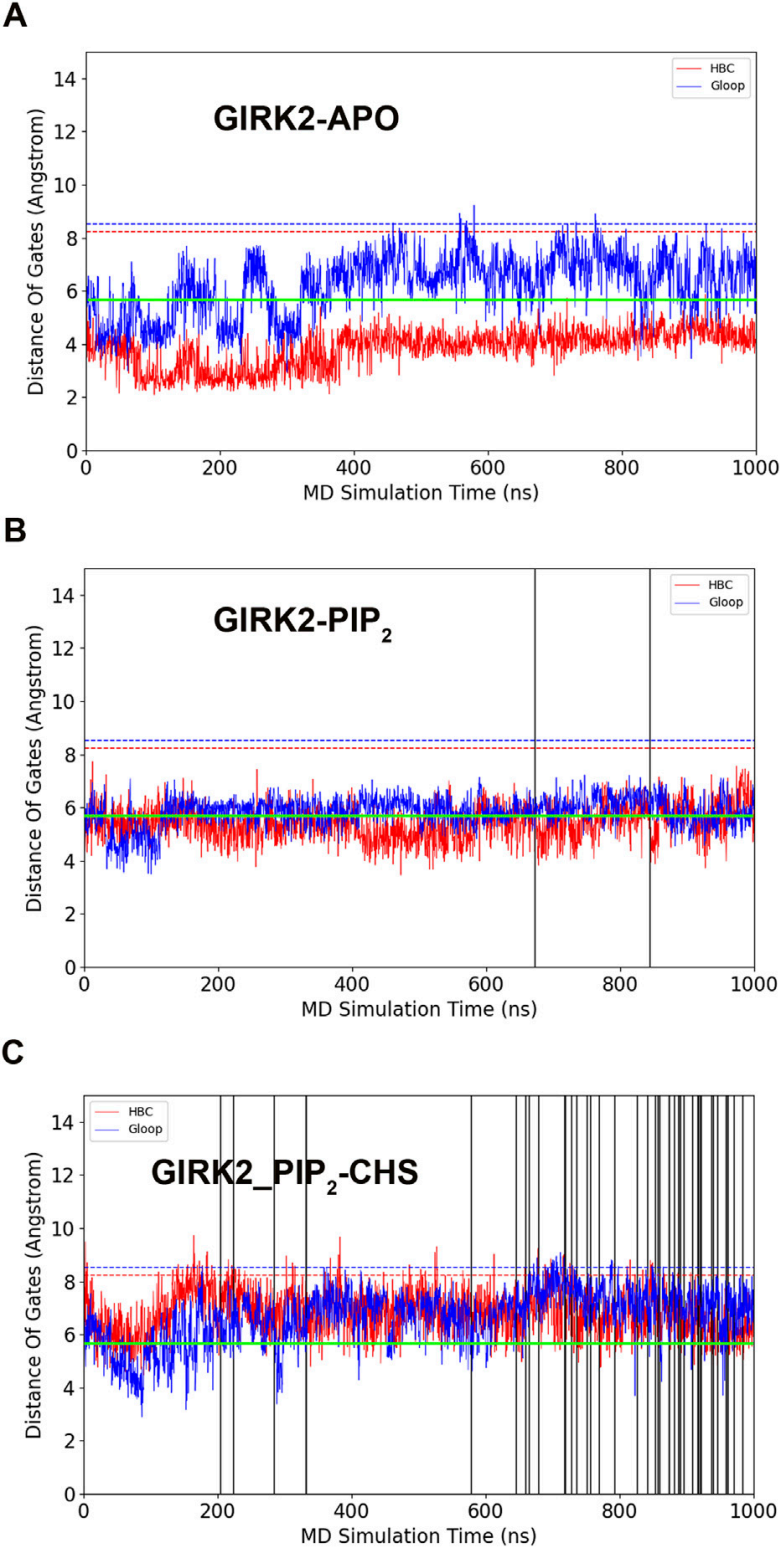
GIRK2 channel gating and K^+^ ion permeation during GMD simulations. **(A)**. GIRK2-APO. **(B)**. GIRK2/PIP_2_. **(C)**. GIRK2/PIP_2_/CHS. Dish lines are gate distances of HBC (red) and G loop (blue) gates in the initial GIRK2 channel structure. Vertical solid lines are the time points for K^+^ ion permeation during the GMD simulations (0 ions for APO, 2 ions for GIRK2/PIP_2_, and 43 ions for GIRK2/PIP_2_/CHS). K^+^ ion permeation was defined as follows: an ion passing through the SF, HBC, and G loop gates from the extracellular to intracellular side along the direction of the electric field. The green lines are the cutoff distance (5.69 Å) for K^+^ ion permeation as shown by Li et al. (2019).

Figure 2 shows K^+^ ion permeation and changes in gate distances (average minimum distances of the HBC and G loop gates) as a function of time during the GMD simulations in each of the three systems. As can be seen, there were no K^+^ ions passing through the GIRK2 channel gates in the APO state. In contrast, 2 and 43 ions passed through the GIRK2 channel gates in the GIRK2/PIP_2_ and GIRK2/PIP_2_/CHS systems during 1µs GMD simulations, respectively.

### Dwell time of K^+^ ions in the GIRK2 channel

**FIGURE 3:**
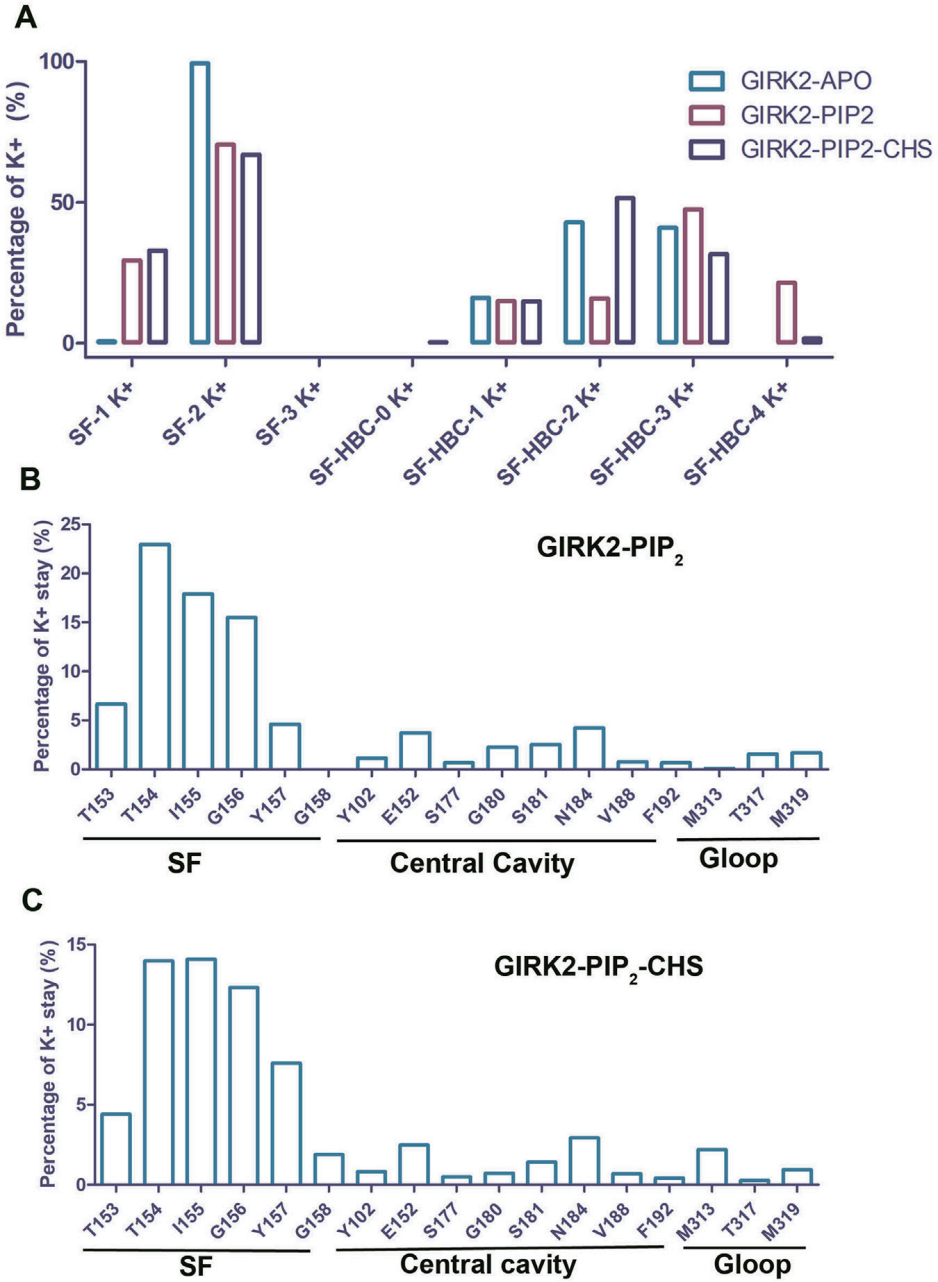
The dwell time of K^+^ ion in the GIRK2 channel. **(A)** Percentage of number of K^+^ ions in selectivity filter (SF) and HBC gate. Percentage of K^+^ dwell time per residue in the channel GIRK2/PIP_2_ **(B)**, and GIRK2/PIP_2_/CHS **(C)**.

To study K^+^ ion permeation, we calculated the percentages of K^+^ ion distribution within the channel for the three systems (Figure 3). During the simulations, the SF (Selectivity Filter) region was predominantly occupied by two K^+^ ions (Apo: 99.33%, PIP_2_: 70.6%, PIP_2_/CHS: 67%). The percentage of the SF region occupied by one K^+^ ion was lower (Apo: 0.64%, PIP_2_: 29.4%, PIP_2_/CHS: 32.96%), while the percentage of the SF region occupied by three K^+^ ions was approximately zero. In the central cavity region between the SF and the HBC (Helix Bundle Crossing) gate of the channel, the percentages of K^+^ ion occupation were as follows for the GIRK2-APO state: 0% (1 ion), 16.16% (2 ions), 42.89% (3 ions), 40.94% (4 ions), and 0% (5 ions). In contrast, for the GIRK2/PIP_2_ system, the percentages were 0% (1 ion), 15.0% (2 ions), 15.97% (3 ions), 47.58% (4 ions), and 21.45% (5 ions). For the GIRK2/PIP_2_/CHS system, the percentages were 0.3% (1 ion), 14.89% (2 ions), 51.56% (3 ions), 31.61% (4 ions), and 1.65% (5 ions) (Figure 3; Supplementary Table S1).

For the K^+^ ion permeation event, a K^+^ ion spent most of its time in the SF region, with 67.62% for the GIRK2/PIP_2_ system and 54.26% for the GIRK2/PIP_2_/CHS system. In contrast, they spent less time in the central cavity and G loop regions: 16.04% and 3.31%, respectively, for the GIRK2/PIP_2_ system; and 9.98% and 3.4%, respectively, for the GIRK2/PIP_2_/CHS system (Figures 3B, C). Fewer K^+^ ions in the central cavity (SF-HBC) in the GIRK2-PIP_2_-CHS system compared to the GIRK2-PIP_2_ system is consistent with increased ion passage and HBC gate opening in the GIRK2-PIP_2_-CHS system (Figure 2).

### GIRK2 conformational changes induced by PIP_2_/CHS

**FIGURE 4:**
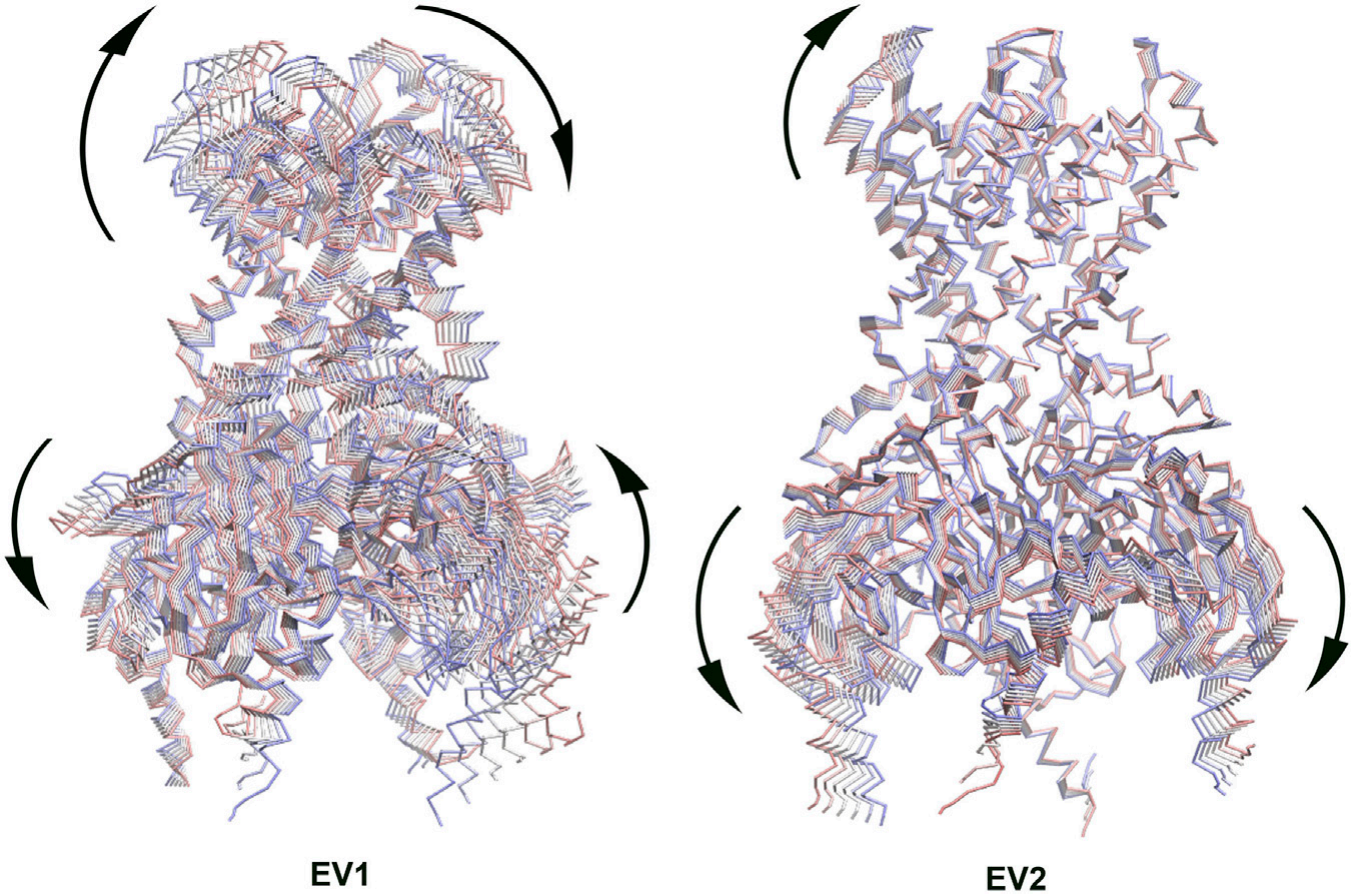
The first eigenvector (EV1) and second eigenvector (EV2) from Combined Principal Component Analysis (PCA) of GIRK2-APO/GIRK2-PIP_2_-CHS based on the MD simulations (200–1,000 ns). The GIRK2 channel structures were shown as Cα traces (six frames colored from red to blue).

Principal Component Analysis (PCA) can be used to extract the collective motions of the proteins from MD trajectories, and also to compare the difference between trajectories between two similar systems, such as, apo and holo states (combined PCA) (Amadei et al., 1993; van Aalten et al., 1995; Amadei et al., 1999; Ceruso et al., 2003). We performed combined PCA analysis based on the concatenated GIRK2/APO and GIRK2/PIP_2_/CHS trajectories (200 ns– 1000 ns) using the GROMACS program (combine two MD simulation trajectories (protein only) (Meng et al., 2012). Figure 4 shows the first and second eigenvectors (EV1 and EV2) from the combined PCA analysis. The major conformational changes between the apo and PIP_2_/CHS bound states are rocking motions between the transmembrane domain and cytosolic domain (EV1), and between cytosolic domains (EV2).

**FIGURE 5:**
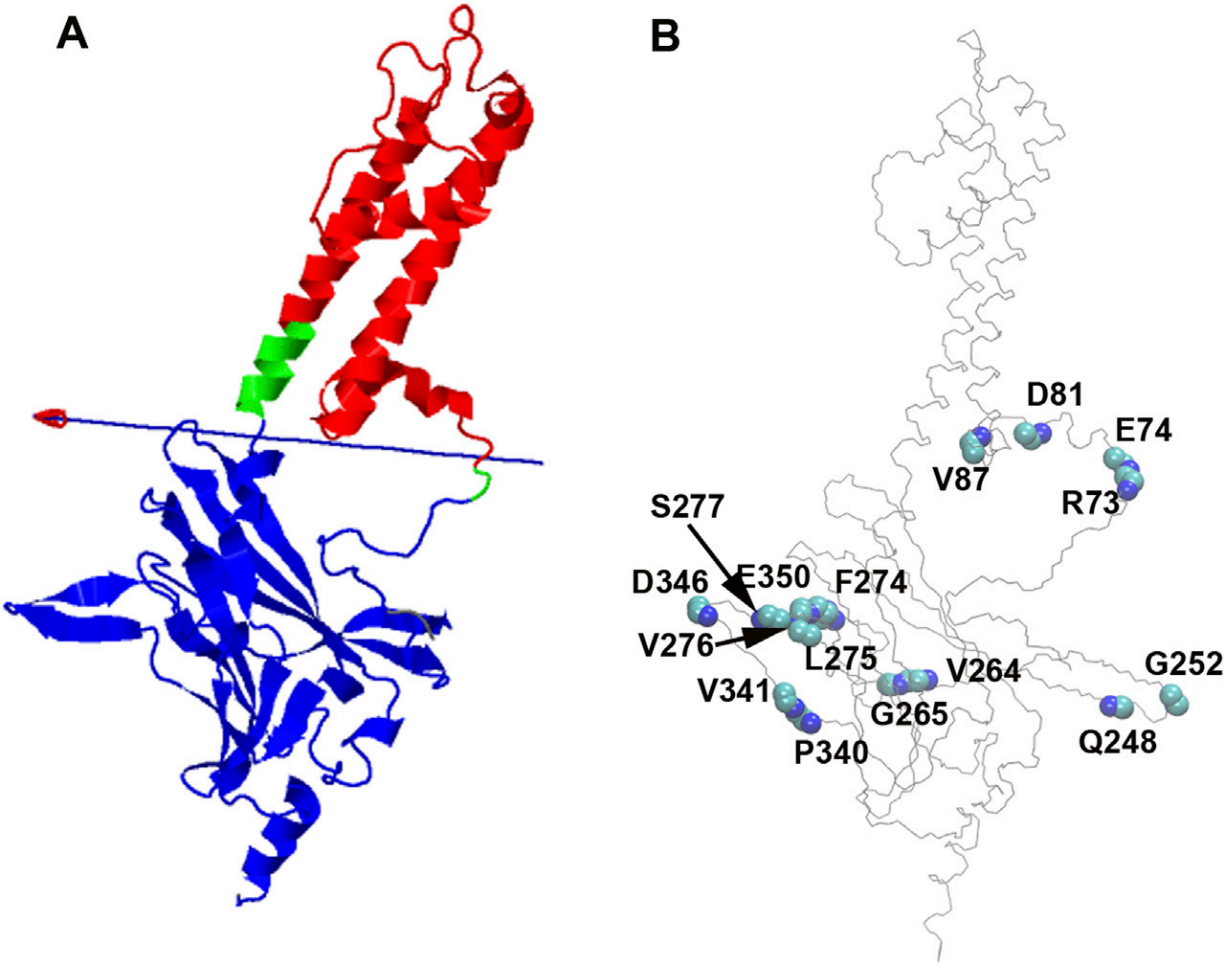
PIP_2_/CHS induced domain motions and backbone conformational changes on GIRK2 channel. **(A)**. Domain and hinge motions identified by DynDom analysis based on PCA (EV1, the first and last frames). Bending residues (hinge, green) are G70-N71, and G189-S196. Domain 1 (cytosolic domain, blue) contains residues 57–70, and 196–380; Domain 2 (transmembrane domain, red) includes residues 71–195. **(B)**. Backbone (phi/psi tortional angles) conformational changes between GIRK2-APO and GIRK2/PIP_2_/CHS during the MD simulations.

DynDom is a program that determines domains, hinge axes and hinge bending residues in proteins where two conformations exist (Lee et al., 2003) (https://dyndom.cmp.uea.ac.uk/dyndom/main.jsp). We analyzed domain and hinge motions on a single subunit of GIRK2 channel using the DynDom webserver. The backbone snapshot structures of GIRK2 from the PCA (EV1, the first and last frames) were used for the DynDom analysis. The hinge residues were identified as βA transition point G70-N71 and the HBC residues G189-S196 (green), which links cytosolic domain (residues 57–70 and 196–380, blue) and transmembrane domain (residues 71–195, red). The bending angle between the two domains is up to 37.6° (Figure 5A), which explains the rocking motions through this hinge region observed in the EV1 from PCA analysis (Figure 4).

We also calculated backbone tortional angles (phi/psi) of GIRK2-APO and GIRK2/PIP_2_/CHS systems during the GMD simulations using the Simulaid program (Mezei, 2010). The residues with the largest difference (average of either phi or psi tortional angles) between the two systems are located in the cytosolic domain which are shown in Figure 5B and listed in Table 1. The dial plots of these phi/psi angle changes as a function of time during the MD simulations are given in Supplementary Figure S3. The center of the plot represents the starting point of the simulation. The blue line depicts the trajectory (starting at the center and ending at the outer circle), while the red line indicates the average of the measured angle.

**TABLE 1:** Selected average phi and psi tortional angles of GIRK1-APO and GIRK2/PIP_2_/CHS systems during MD simulations. Simulaid program was used for the calculations.

	GIRK2-APO		GIRK2-PIP_2_-CHS	
	Phi	Psi	Phi	Psi
R73	–82.96	21.52	–91.88	177.68
E74	–115.3	29.22	58.29	48.09
D81	–126.51	29.48	–85.65	23.23
V87	–117.83	–39.21	–64.21	–43.2
Q248	–139.19	20.37	–115.96	143.05
G252	–172.83	–178.71	88.54	–0.2
V264	–123.78	5.26	–111.43	140.07
G265	157.62	–4.85	68.72	14.37
F274	–87.17	–32.17	–127.73	82.26
L275	60.01	11.88	–84.32	109.06
V276	–73.13	–24.51	–123.59	–69.22
S277	–131.25	150.04	–85.57	158.99
P340	–58.73	144.48	–72.02	5.86
V341	–122.15	–25.95	–89.06	129.98
D346	53.42	25.93	–0.51	50.45
E350	–134.99	144.18	–83.2	133.66

Next we embark to identify in an unbiased manner the major interaction changes (salt bridges, hydrogen bonds, hydrophobic interactions and correlation pairs) between the completely non-permeable (GIRK2-APO) system and the fully permeable (GIRK2-PIP2-CHS) system. We reserve interpretation of these results to the Discussion section of the manuscript.

### Salt bridge interaction network changes in GIRK2 by PIP_2_/CHS

**FIGURE 6:**
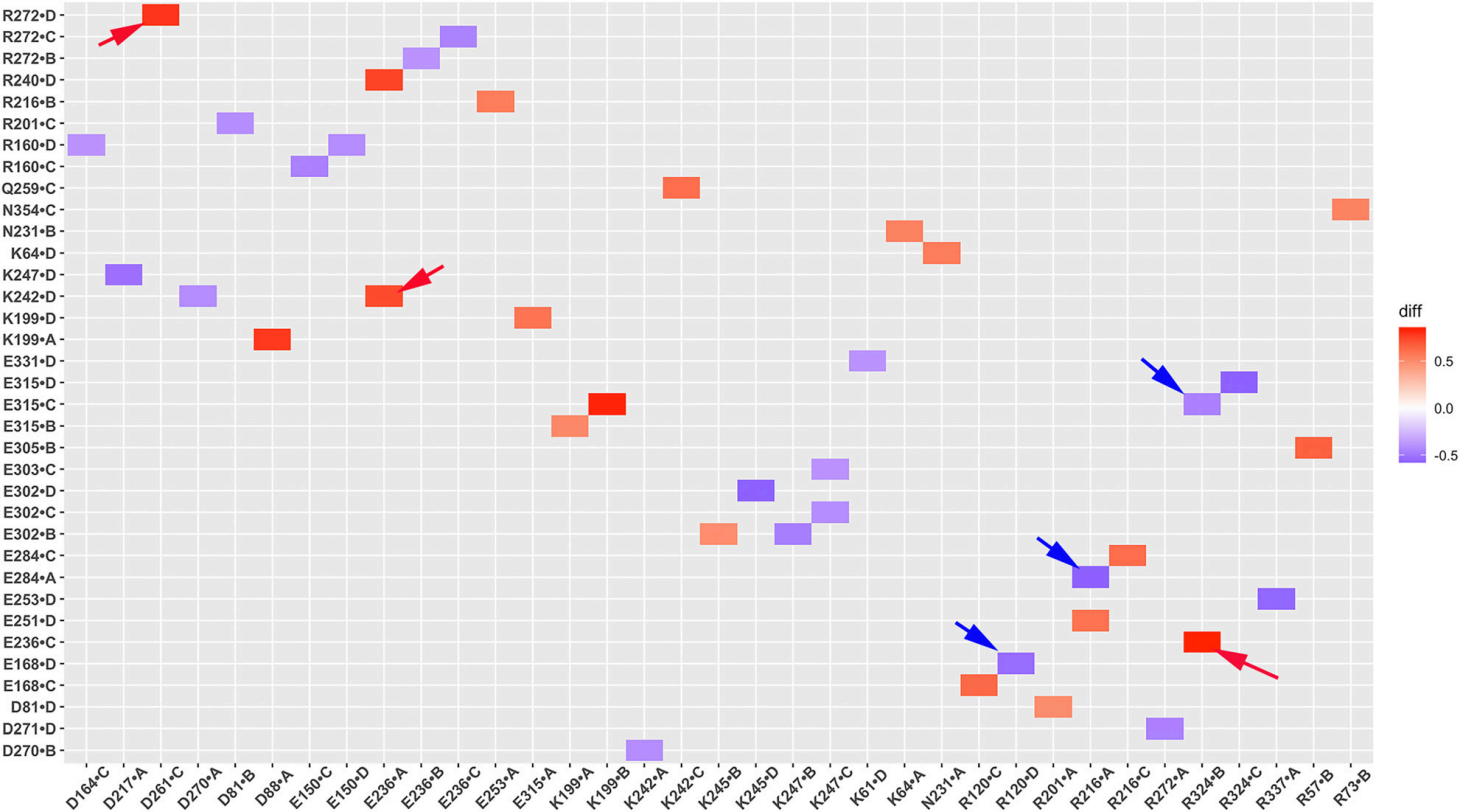
A heatmap plot of salt bridge pairs (Red: salt bridge formed; Blue: disrupted in GIRK2-APO system). Comparison of key salt bridge interactions between GIRK2-APO and GIRK2-PIP_2_-CHS based on MD simulations (200–1,000 ns). Selected salt bridge pairs are marked with arrows to show in the GIRK2 structure for Figure 7.

Salt bridge interaction network interactions are important for protein function. To further understand critical salt bridge interactions involved in GIRK2 channel activation, we conducted an analysis of the salt bridge interaction network using the Simulaid program (Mezei, 2010). We quantified the formation of salt bridge pairs as a fraction of the GMD trajectories in both the APO and PIP_2_/CHS-bound systems, and then compared the differences between these two systems. The salt bridge pairs exhibiting the most significant differences in fraction (percentage of salt bridge formation) during the simulations were identified for detailed analysis (See Supplementary Table S2). Figure 6 illustrates these differences in fraction through a heatmap plot, where red squares indicate the formation of salt bridges in the GIRK2-APO system, while blue squares indicate the formation of salt bridges in the GIRK2/PIP_2_/CHS system.

**FIGURE 7:**
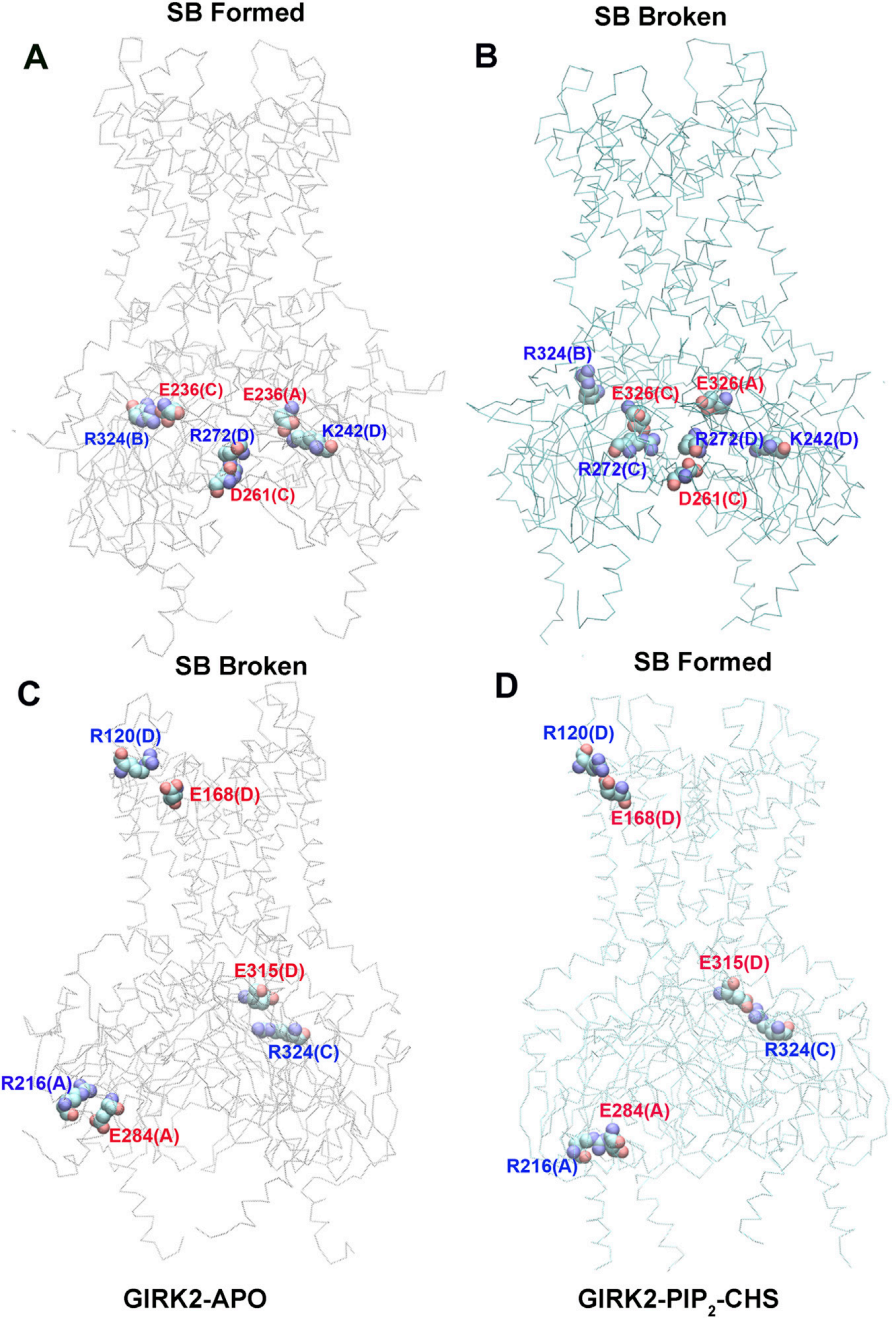
Selected key different salt bridge residues in GIRK-APO, and GIRK2-PIP_2_-CHS. **(A)**. Salt bridge pairs, E236(C)-R324(B), R272(D)-D261(C), and E236(A)-K242(D) formed in GIRK2-APO; and **(B)**. broken in GIRK2/PIP_2_/CHS. **(C)**. Salt bridge pairs, R120(D)-E168(D), R216(A)-E284(A), and E315(D)-R324(C) broken in GIRK2-APO; **(D)**. formed in GIRK2/PIP_2_/CHS. The last frame of GIRK2-APO and GIRK2/PIP_2_/CHS from 1µs MD simulations are used in this figure.

Figure 7 shows selected salt bridge pairs, E236(C)-R324(B), R272(D)-D261(C), and E236(A)-K242(D) formed in the GIRK2-APO and broken in the GIRK2/PIP_2_/CHS systems. While the salt bridge pairs, R120(D)-E168(D), R216(A)-E284(A), and E315(D)-R324(C) are broken in the GIRK2-APO, and formed in the GIRK2/PIP_2_/CHS systems. Supplementary Figure S4 shows the selected salt bridge pair distances as a function of time for GIRK2-APO and GIRK2/PIP_2_/CHS systems during 1µs GMD simulations.

Residue E236 (CD loop) points to the inside of the tunnel and forms salt bridges with R272 (EGF) in the GIRK2/PIP_2_/CHS system, while it forms salt bridges with residues R324 (near the G loop), R272, R240, and K242 (βD) in the GIRK2-APO system. Residue E315 (G loop) forms stronger salt bridges with R324 (G loop, adjacent subunit) in the GIRK2/PIP_2_/CHS system, while it forms salt bridges with K199 (βL) in the GIRK2-APO system. A salt bridge forms between D88 (S-HLX) and K199 in the GIRK2-APO system, but weakens in the GIRK2/PIP_2_/CHS system. Residue R57 forms a strong salt bridge with E311 in both systems, while it forms a stronger salt bridge with E305 in the GIRK2-APO system compared to the GIRK2/PIP_2_/CHS system. Residue R272 forms a stronger salt bridge interaction with D261 and D271 in the GIRK2-APO system, while it forms a stronger salt bridge interaction with E236 in the GIRK2/PIP_2_/CHS system. K247 shows increased salt bridge interactions with residues E302 and E303 in the GIRK2/PIP_2_/CHS system. Residue E150 (pore helix) forms a stronger salt bridge interaction with R160 (SF) in the GIRK2-APO system compared to the GIRK2/PIP_2_/CHS system (Supplementary Figure S5).

### Hydrogen bond interaction network changes in GIRK2 by PIP_2_/CHS

**FIGURE 8:**
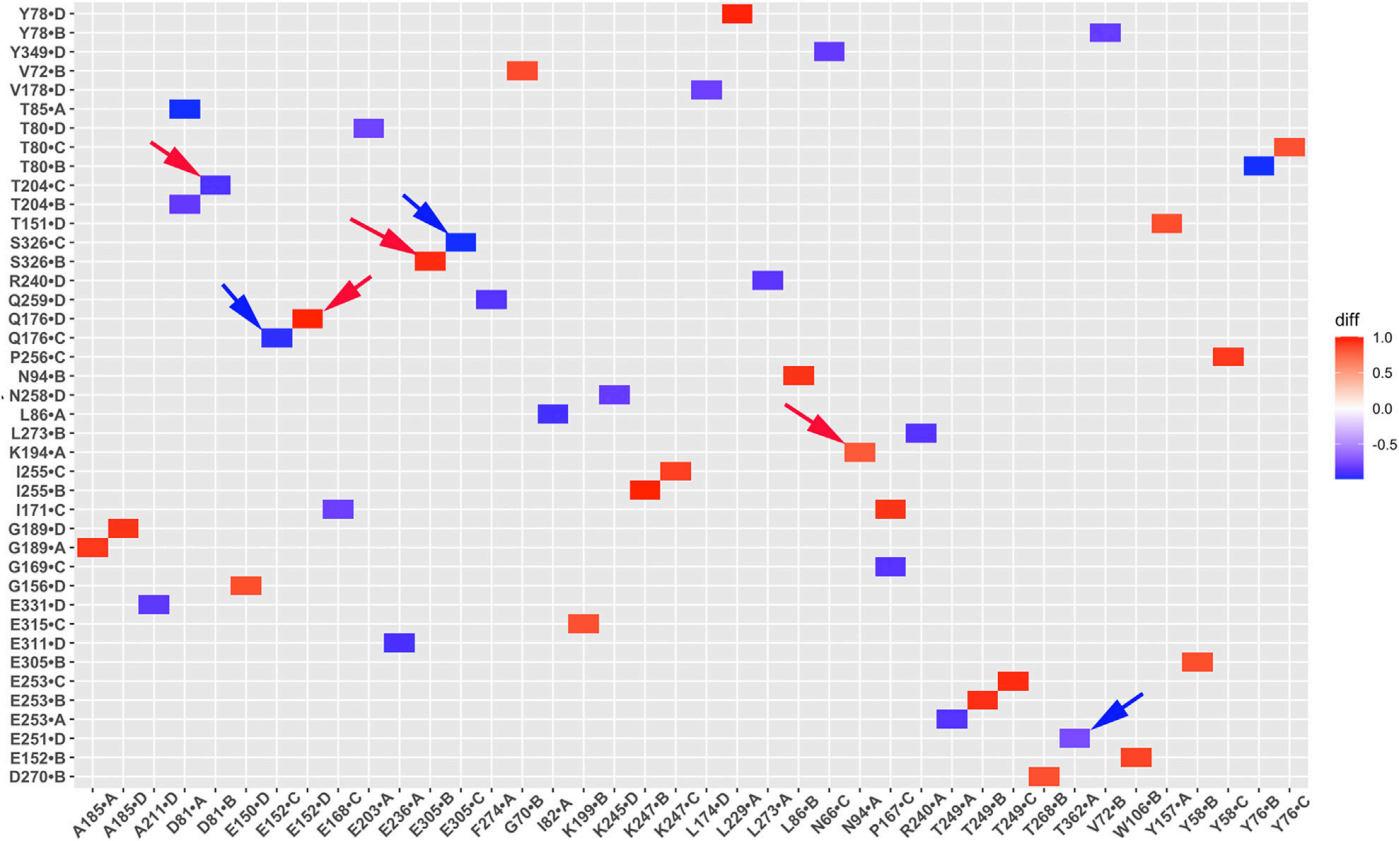
A heatmap plot of hydrogen bond pairs (Red: HB formed; Blue: HB disrupted in GIRK2-APO system). Comparison of key HB interactions between GIRK2-APO and GIRK2-PIP_2_-CHS based on MD simulations (200–1,000 ns). Selected HB pairs are marked with arrows to show in the GIRK2 structure for Figure 9.

**FIGURE 9:**
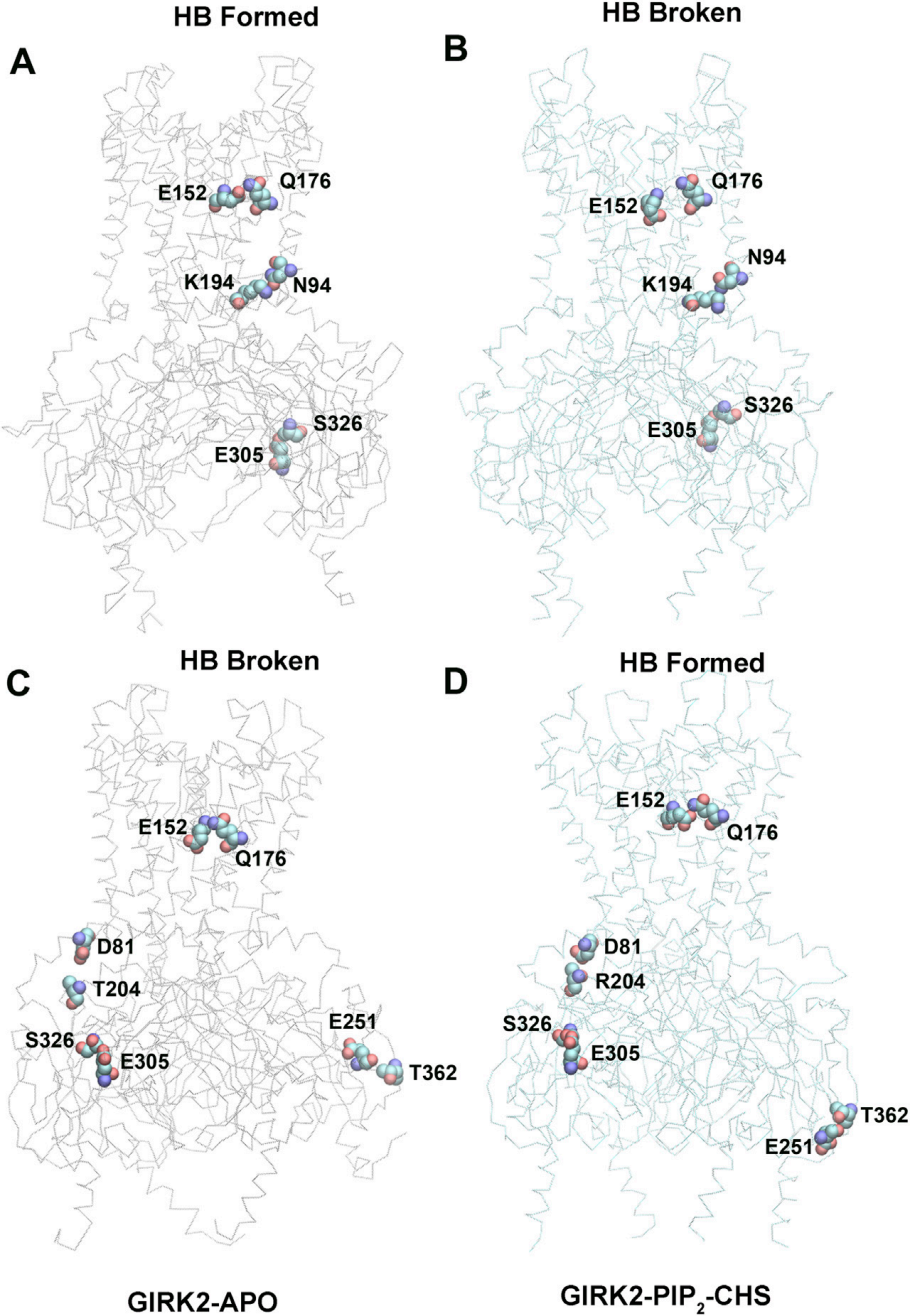
Selected key different HB residues in GIRK-APO, and GIRK2-PIP_2_-CHS. **(A)**. HB pairs, E152(D)-Q176(D), K194(A)-N94(A), E305(B)-S326(B) formed in GIRK2-APO; and **(B)**. broken in GIRK2/PIP_2_/CHS. **(C)**. HB pairs, D81(C)-T204(B), S326(C)-E305(C), E251(D)-T362(A), and E152(C)-Q176(C) broken in GIRK2-APO; **(D)**. formed in GIRK2/PIP_2_/CHS. The last frame of GIRK2-APO and GIRK2/PIP_2_/CHS from 1µs MD simulations are used in this figure.

Hydrogen bond interactions play a crucial role in protein structure and function. For instance, they contribute to the formation of key protein secondary structures like α helices and β sheets. We performed systematically hydrogen bond network analysis based on GMD simulation trajectories, and compared the hydrogen bond changes in fraction (percentage of hydrogen bond formation) between GIRK2-APO and GIRK2/PIP_2_/CHS systems using the Simulaid program (Mezei, 2010). Figure 8 illustrates the variation in hydrogen bond interaction pairs between the GIRK2-APO and GIRK2/PIP_2_/CHS systems using a heatmap plot. In this figure, red squares indicate an increase in the fraction of hydrogen bond pairs in the GIRK2-APO system, while blue squares represent an increase in the fraction of hydrogen bond pairs in the GIRK2/PIP_2_/CHS system. Figures 9A, B display a fraction of hydrogen bond pairs higher in the GIRK2-APO, and lower in the GIRK2/PIP_2_/CHS systems, respectively (E152(D)-Q176(D), K194-N94, and E305-S326), whereas Figures 9C, D exhibits a fraction of hydrogen bond pairs higher in the GIRK2/PIP_2_/CHS, and lower in the GIRK2-APO systems (E152(C)-Q176(C), D81-T204, S326-E305, and E251-T362). The detailed hydrogen bond fraction information for selected key different hydrogen bond residues in GIRK-APO and GIRK2-PIP_2_-CHS is given in Supplementary Table S3. Supplementary Figure S6 shows the selected HB pair distances as a function of simulation time for GIRK2-APO and GIRK2/PIP2/CHS systems during 1µs GMD simulations.

E315 (G loop)-H233(CD loop) shows increased hydrogen bonds formation in GIRK2/PIP_2_/CHS system compared to the GIRK2-Apo system. There are some hydrogen bonds between β-loop/CD-loop and S-HLX, for examples, T204 (β-loop)-D81(S-HLX) and E203 (β-loop)-T80(S-HLX) shows increased HB formation, while L229(CD loop)-Y78(S-HLX) and Q197 (β-loop)-V87(S-HLX) shows decreased HB formation in the GIRK2/PIP_2_/CHS system compared to the GIRK2-APO systems. CD loop residues, N231 and D228 form HB with βA residues, H68 (increased in GIRK2/PIP2/CHS) and H69 (decreased in GIRK2-APO), respectively. Residue E152(PH) shows increased HB formation with residues W106 (TM1) and Q176 (TM2) in the GIRK2-APO system. Residue pairs, Y349 (βM)-N66 (n-ter), R204 (βD)-L273 (EGF), E251 (DE loop)-T362 (βN), and S277 (EGF)-Y58 (n-ter) show increased HB formation in GIRK2/PIP_2_/CHS systems, while Y157(SF)-T151(PH) shows increased HB formation in the GIRK2-APO system (Supplementary Figure S7).

### Hydrophobic interaction network changes in GIRK2 by PIP_2_/CHS

**FIGURE 10:**
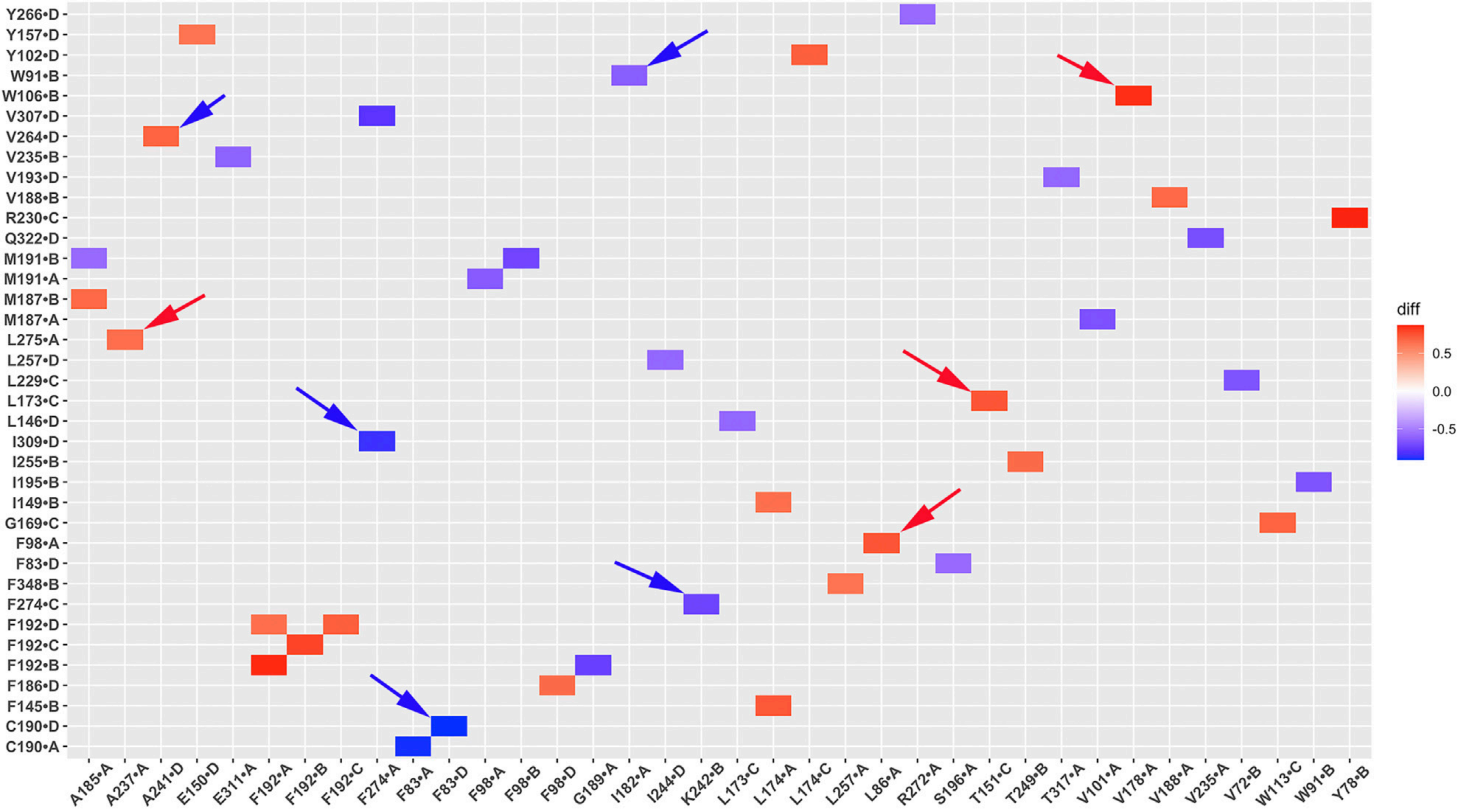
A heatmap plot of hydrophobic pairs (Red: HP formed; Blue: HP disrupted in GIRK2-APO system). Comparison of key HP interactions between GIRK2-APO and GIRK2-PIP_2_-CHS based on MD simulations (200–1,000 ns). Selected HP pairs are marked with arrows to show in the GIRK2 structure for Figure 11.

Unlike salt bridges and hydrogen bonds, which rely on electrostatic interactions between polar residues, hydrophobic interactions are driven by the interactions between non-polar residues and water molecules. This drives non-polar residues to associate with each other in energetically favorable states. Hydrophobic interactions play a critical role in protein structure and function. To investigate the significance of hydrophobic residues in GIRK2 receptor activation, we conducted a systematic analysis of hydrophobic interaction networks and calculated the differences in fraction for each hydrophobic residue pair between the GIRK2-APO and GIRK2/PIP_2_/CHS systems using Simulaid program (Mezei, 2010). Figure 10 illustrates the variation in hydrophobic interaction pairs between the GIRK2-APO and GIRK2/PIP_2_/CHS systems using a heatmap plot. In this visualization, red squares indicate an increase in the fraction of hydrophobic interaction pairs in the GIRK2-APO system, while blue squares represent an increase in the fraction of hydrophobic interaction pairs in the GIRK2/PIP_2_/CHS system. Supplementary Table S4 presents hydrophobic pairs exhibiting significant differences in fractions between the GIRK2-APO and GIRK2/PIP_2_/CHS systems based on MD simulation trajectories.

**FIGURE 11:**
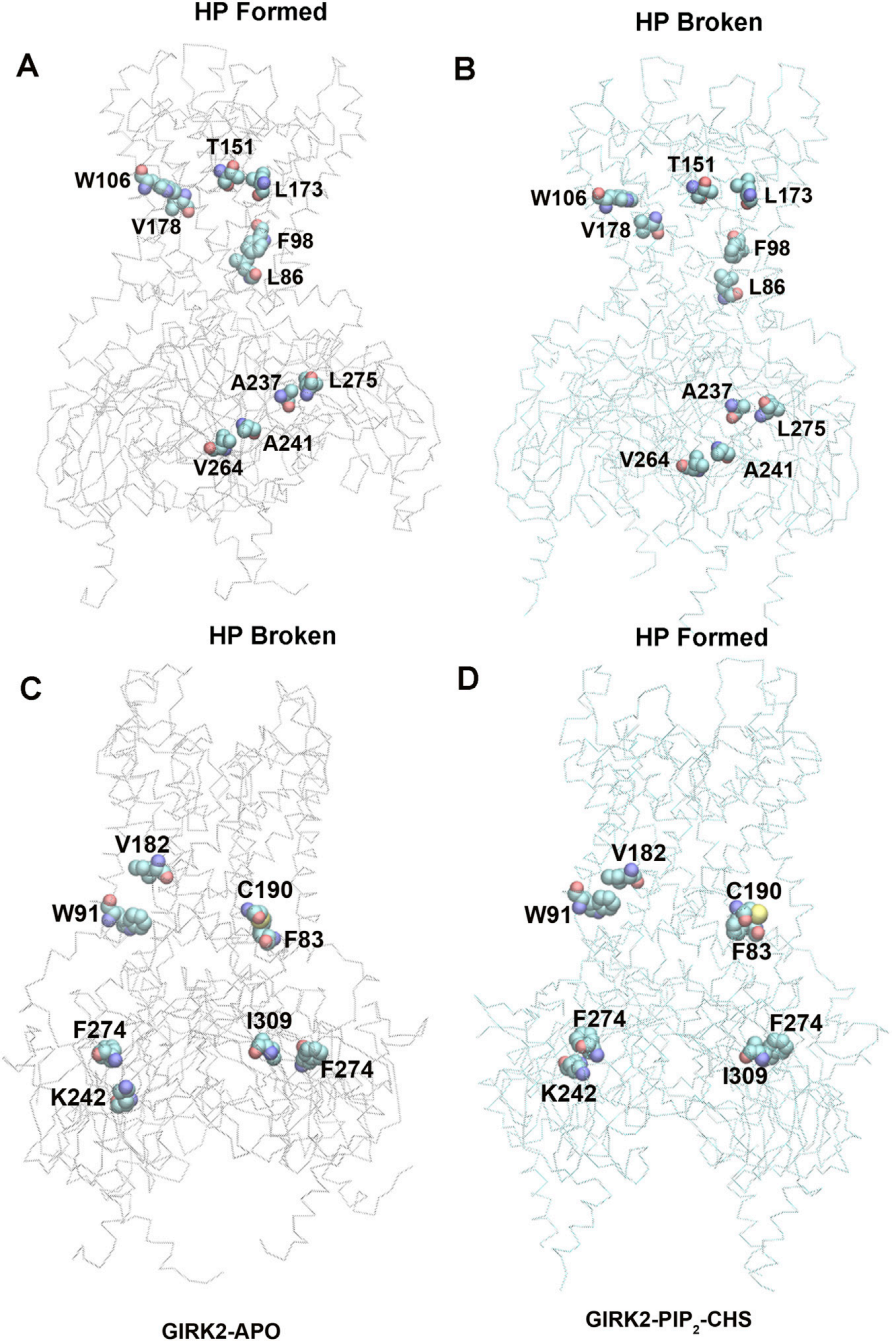
Selected key different HP residues in GIRK-APO, and GIRK2-PIP_2_-CHS. **(A)**. HP pairs, W106-V178, T151-L173, F98-L86, A237-L275, and A241-V264 formed in GIRK2-APO; and **(B)**. broken in GIRK2/PIP_2_/CHS. **(C)**. HB pairs, V182-W91, C190-F83, F274-K242, and F274-I309 broken in GIRK2-APO; **(D)**. formed in GIRK2/PIP_2_/CHS. The last frame of GIRK2-APO and GIRK2/PIP_2_/CHS from 1µs MD simulations are used in this figure.

Figures 11A, B show some selected hydrophobic pairs formed in the GIRK2-APO but absent in the GIRK2/PIP_2_/CHS systems, these are W106-V178, T151-L173, F98-L86, A237-L275 and A241-V264. Figures 11C, D show some selected hydrophobic pairs absent in the GIRK2-APO, but formed in the GIRK2/PIP_2_/CHS systems, these are V182-W91, C190-F83, F274-K242 and F274-I309. Supplementary Figure S8 shows the selected HP pair distances as function of time for GIRK2-APO and GIRK2/PIP_2_/CHS systems during 1µs MD simulations.

Residue pairs, F192(HBC)-F192(HBC, adjacent subunit), M187 (TM2)-A185 (TM2, adjacent subunit), F186 (TM2)-F98 (TM1), I149(PH)-L173 (TM2), L172 (TM2)-I112 (TM1), and Y349 (βM)-P256 (βE) show increased HP interaction formation in the GIRK2-APO system. The residue pairs, C190 (TM2)-F83(S-HLX), S196 (TM2)-F83(S-HLX), F186 (TM2)-I82(S-HLX), M187 (TM2)-V101 (TM1), M191 (TM2)-F98 (TM1), I195 (TM2)-W91 (TM1), V178 (TM2)-F98 (TM1), I309 (βH)-F274 (EGF), F274 (EGF)-K242 (βD), Y353 (βM)-V206(B-loop) show increased HP interaction formation in the GIKR2/PIP_2_/CHS system (Supplementary Figure S9).

### Residue pair correlation network changes in GIRK2 by PIP_2_/CHS

**FIGURE 12:**
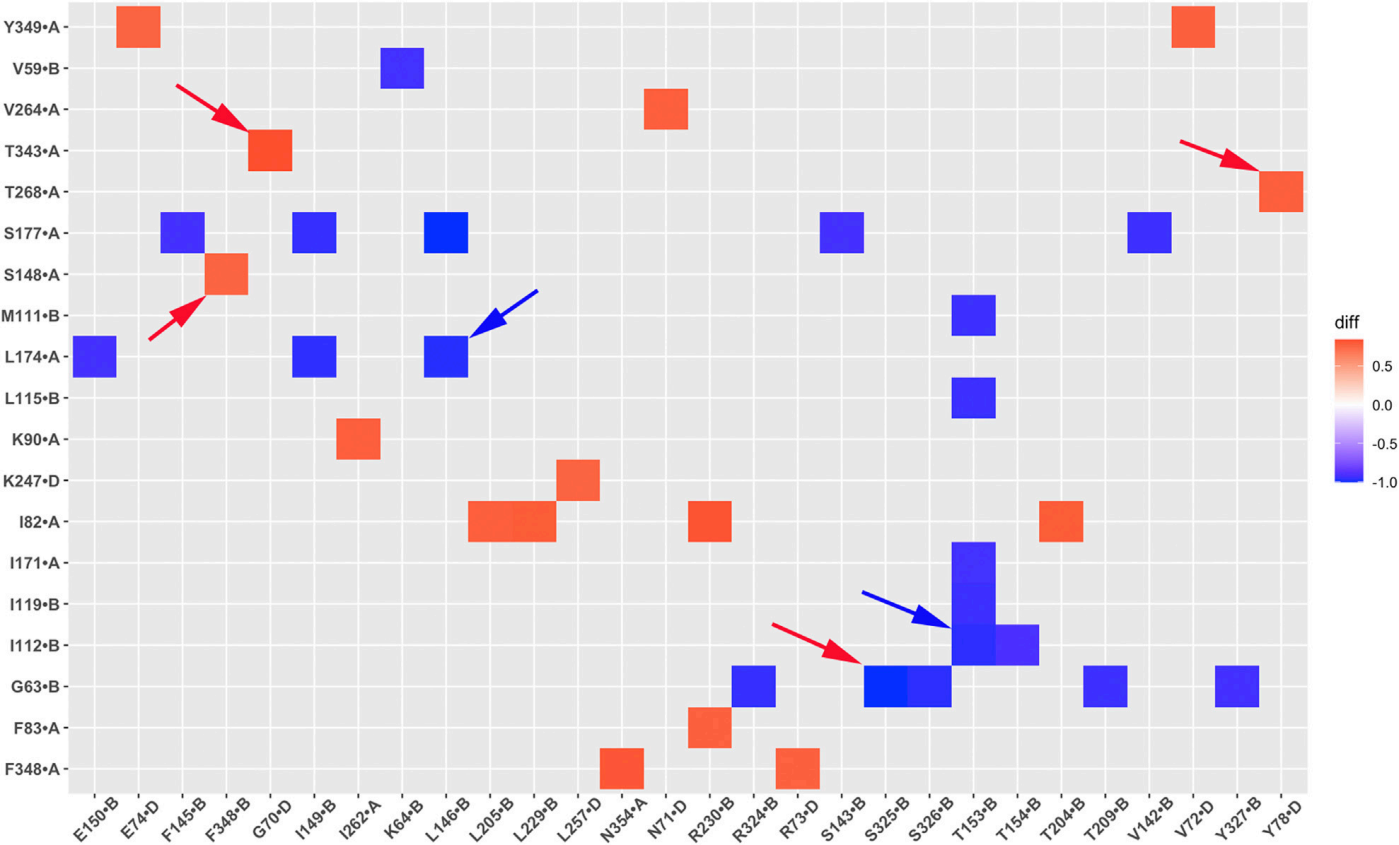
A heatmap plot of residue correlation pairs (Red: positive correlation; Blue: negative correlation in GIRK2-APO system). Comparison of key residue correlations between GIRK2-APO and GIRK2-PIP_2_-CHS based on MD simulations (200–1,000 ns). Selected correlated residue pairs are marked with arrows to show in the GIRK2 structure for Figure 13.

The correlation between residue pairs reflects protein dynamic characteristics and can offer valuable insights into understanding channel activation. We performed systematically analysis of residue pair correlation and compared the difference in fraction between the GIRK2-APO and the GIRK2/PIP_2_/CHS systems. Figure 12 illustrates the variation in correlations of residue pairs between the GIRK2-APO and GIRK2/PIP_2_/CHS systems using a heatmap plot. In this visualization, red squares indicate an increase in the fraction of positive correlations of residue pairs in the GIRK2-APO system, while blue squares represent an increase in the fraction of positive correlations of residue pairs in the GIRK2/PIP_2_/CHS system.

**FIGURE 13:**
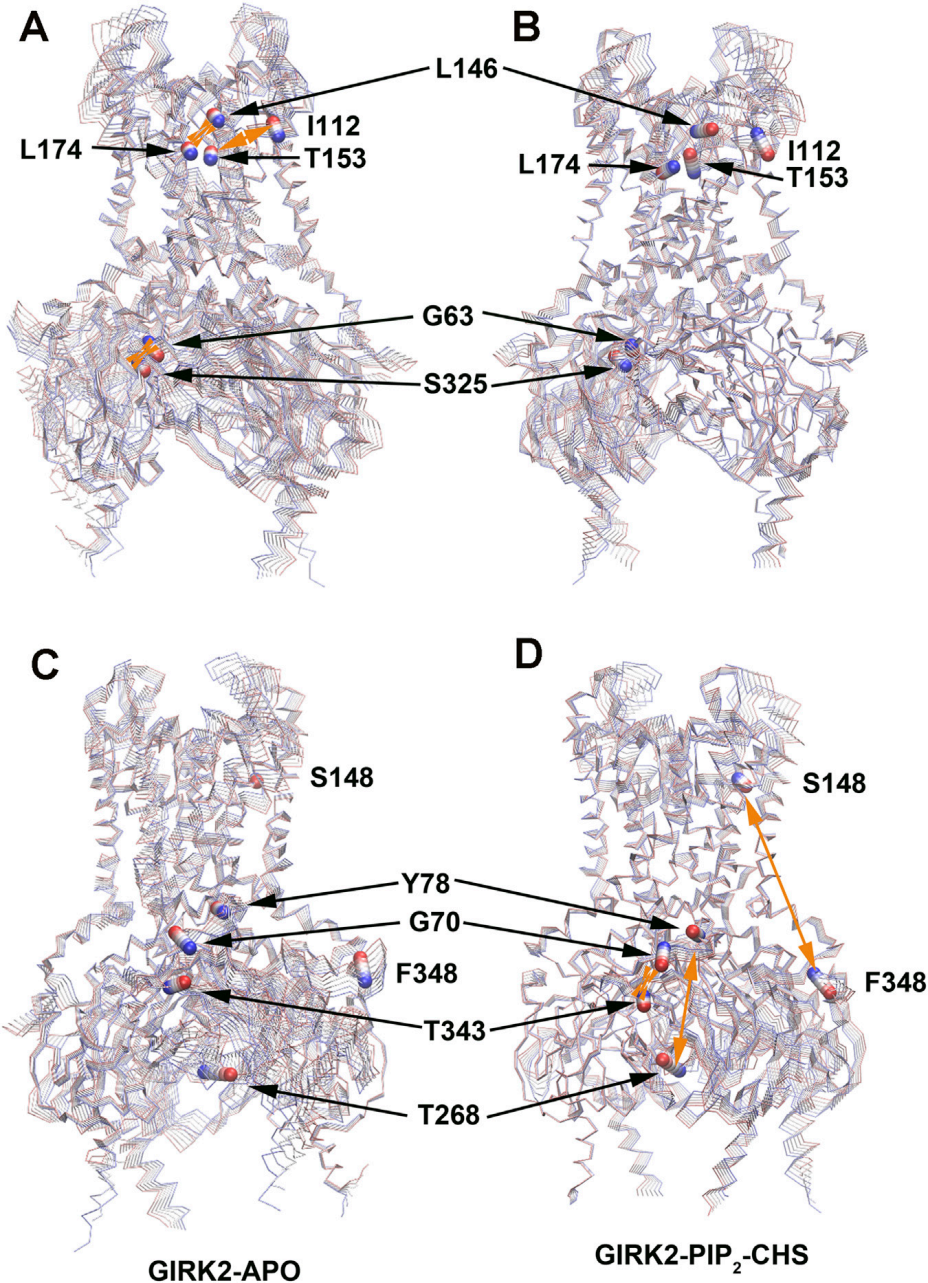
Selected correlated residues in GIRK-APO, and GIRK2-PIP2-CHS. **(A)** correlated residue pairs, L146-L174, I112-T153 and G63-S325 positively correlated in GIRK2-APO; and **(B)** negatively correlated in GIRK2/PIP_2_/CHS. **(C)** correlated residue pairs, S148-F348, G70-T343, and Y78-T268 negatively correlated in GIRK2-APO; **(D)** positively correlated in GIRK2/PIP_2_/CHS. The first eigenvector (EV1) of GIRK2-APO and GIRK2/PIP_2_/CHS from MD simulations are used in this figure. Color coding (blue to red) on Cα atoms (balls) of selected residues shows a transition of the Cα atom movement in the EV1.

Table 2 lists selected correlated residue pairs that show significant differences between the GIRK2-APO and GIRK2/PIP_2_/CHS systems. Figures 13A, B show some selected positively correlated residue pairs in the GIRK2-APO but negatively correlated in the GIRK2/PIP_2_/CHS systems, these are L146-L174, I112-T153 and G63-S325. Figures 13C, D show some selected negatively correlated residue pairs in the GIRK2-APO, but positively correlated in the GIRK2/PIP_2_/CHS systems, these are S148-F348, G70-T343, and Y78-T268.

**TABLE 2:** Selected correlated residue pairs show the difference between GIRK2-APO and GIRK2/PIP_2_/CHS.

Residue pair	GIRK2-APO Correlation coefficient	GIRK2-PIP_2_-CHS Correlation coefficient	Correlation coefficient difference
G63-S325	0.6885	–0.3178	–1.0063
L174-L146	0.5050	–0.4728	–0.9778
I112-T153	0.5452	–0.4264	–0.9716
T343-G70	–0.1971	0.6466	0.8437
T268-Y78	–0.3882	0.3976	0.7858
S148-F348	–0.2697	0.4942	0.7639

Residue pairs Y78(S-HLX)-Y159(SF), D81(S-HLX)-L205(B-loop)/F93 (TM1), I82(S-HLX)-Y76(S-HLX, adjacent subunit)/D352 (βM), L89(S-HLX)-I175 (TM2)/F192(HBC)/R225 (βG)/L279 (EGF), R92 (TM1)-A316 (G loop), S208-E315 (G loop), I306 (βH)-E352 (βM), N258 (βE)-F348 (βM) show increased positive correlations in the GIRK2/PIP_2_/CHS system. While the residue pairs G63(N-ter)-T209 (βB)/E305 (βH)/H357 (βN), I116 (TM1)-L107 (TM1)/F145(PH)/T153(PH), Y118 (TM1)-L174 (TM2), I171 (TM2)-I155(SF)/G158(SF)/Y159(SF), Y360 (βN)-S250 (βD), F254 (βE)-H357 (βN) show increased negative correlations in the GIRK2/PIP_2_/CHS system compared to the GIRK2-APO system (Supplementary Figure S10).

### CHS binding sites and key interacting residues in GIRK2 channel

**FIGURE 14:**
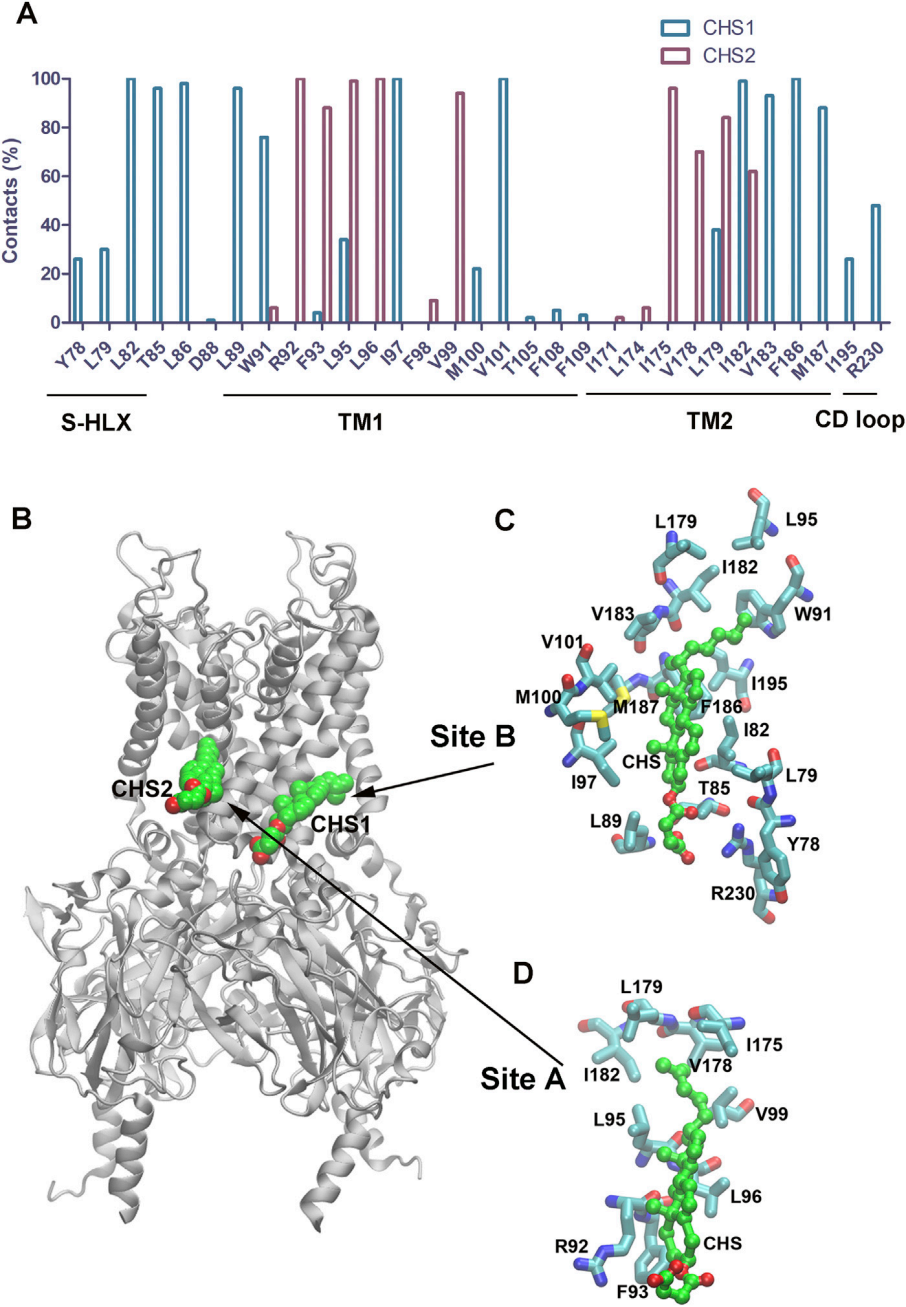
The binding site residues in GIRK2 interact with CHS. **(A)**. Percentage contacts of the binding site residues in GIRK2 interactions with CHS during MD simulations (200–1,000 ns). **(B).** Binding sites for CHS1 and CHS2 in GIRK2. **(C)**. Key CHS interacting residues in the CHS1 binding site B. **(D).** Key CHS interacting residues in the CHS2 binding site A. The snapshot was taken from the frame (265 ns) of the simulations.

We also characterized the two binding sites for CHS in the GIRK2 channel by calculating percentage of key binding residue contacts using the Simulaid program (Mezei, 2010) to scan the MD simulation trajectory (200ns–1000 ns) of the GIRK2/PIP_2_/CHS system. Figure 14A shows percentage of contacts for the binding site residues in GIRK2 interactions with CHS during 1µs MD simulations. In binding site B, the CHS molecule interacts with residues in the slide helix (S-HLX), Y78, L79, L82, T85, and T86; TM1: W91-F109; TM2: L179-I195; and CD loop: R230. While in binding site A, the CHS interacts with residues in TM1: W91-V99; and TM2: I171-I182. The detailed interactions of the key residues in site B and site A are shown in Figures 14C, D, respectively.

### CHS enhances PIP_2_ and GIRK2 channel interactions

**FIGURE 15:**
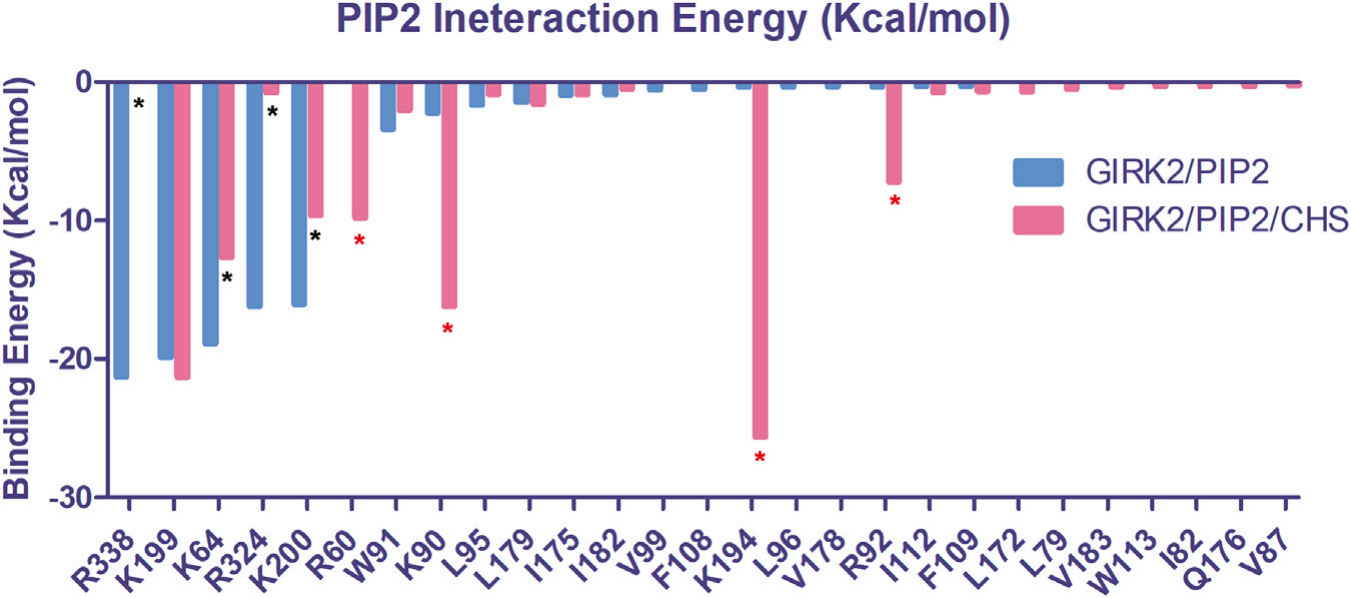
The critical PIP_2_ interacting residues in the binding site of GIRK2 channel. Interaction energies for each residue were calculated using the MM-GBSA method of the Amber program. The interacting residues are colored blue for the GIRK2/PIP_2_ (Total interaction energy: −80.70 kcal/mol) and red for the GIRK2/PIP_2_/CHS (Total interaction energy: −98.97 kcal/mol) systems. Asterisks (*) denote significant changes seen in specific residue interactions with PIP_2_ in the conducting system induced by the presence of CHS (blue: reductions, red: increases).

Using the MM-GBSA method in the Amber program, we calculated the binding free energy interactions between PIP_2_ and the channel for the GIRK2/PIP_2_ and GIRK2/PIP_2_/CHS systems. The binding free energies for PIP_2_ with the channel are −80.70 kcal/mol and −98.97 kcal/mol for the GIRK2/PIP_2_ and GIRK2/PIP_2_/CHS systems, respectively, indicating that CHS binding enhances the interactions between PIP_2_ and the channel. Figure 15 and Supplementary Tables S6, S7 show each individual binding site residue’s contribution to the total binding free energy for both systems. Thus, our data suggest that an overall enhancement of ∼20 kcal/mol enabled by CHS is sufficient to increase permeation ∼20-fold, from 2 ion in the GIRK2/PIP_2_ system to 43 ions in the GIRK2/PIP_2_/CHS system.

### PIP_2_ alters the internal electric field in the GIRK2 channel and facilitates ion permeability

**FIGURE 16:**
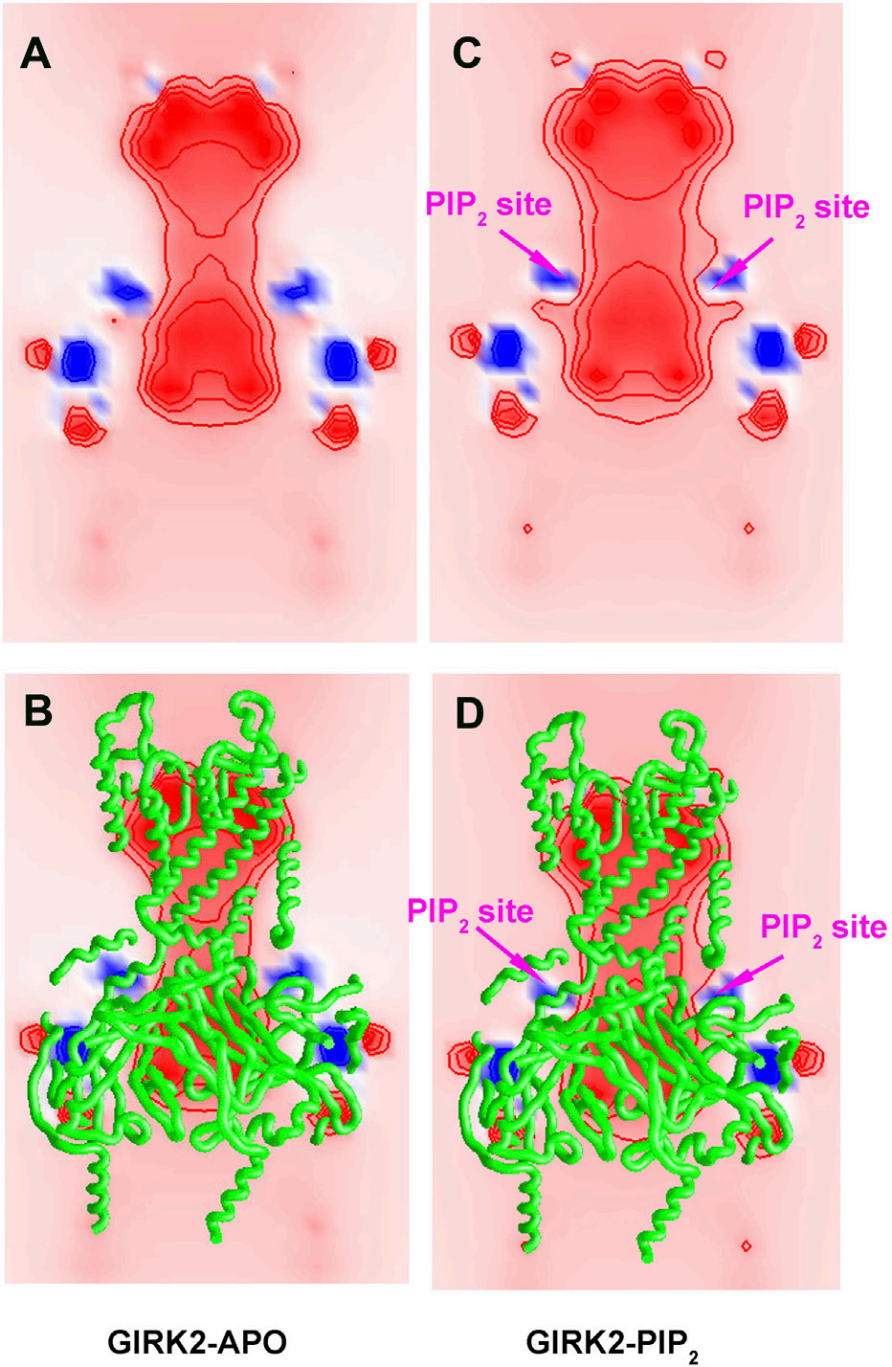
Electrostatic potential maps for GIRK2-APO and GIRK2-PIP_2_. A cross-section map of GIRK2-APO **(A, B)**, and GIRK2/PIP_2_ **(C, D)** to show electrostatic potential distribution in the channel. The negative and positive electrostatic potential regions are colored by red and blue, respectively. The values of the electrostatic potential contours are ± 10, ± 20, and ± 40 kT/e, from outside to inside. The Grasp program was used for electrostatic potential calculations and figure generation.

Since PIP_2_ carries negative charges (-5e each), a total of −20e charges (from 4 PIP_2_ molecules) are added to the GIRK2 channel, which should alter the internal electric field in the channel. To better understand how PIP_2_ alters the internal electric field of the GIRK2 channel, we calculated the electrostatic potential for the GIRK2-APO and GIRK2-PIP_2_ systems using the Grasp software (Nicholls et al., 1991). Figure 16 shows clearly the difference between GIRK2 with and without the presence of PIP_2_. The results show that there is a strong negative electrostatic potential in both the pore and cytosolic domains, but a relatively weaker negative electrostatic potential in the HBC region, creating an energy barrier for K^+^ ion passage. Upon PIP_2_ binding, the negative electrostatic potential increases in the HBC region, thereby reducing the electrostatic energy barrier for K^+^ ion permeation.

## Discussion

We conducted a study to investigate the molecular basis of GIRK2 activation by PIP_2_/CHS using MD simulations. Three systems, namely, GIRK2-APO, GIRK2/PIP_2_, and GIRK2/PIP_2_/CHS, underwent 1µs MD simulations, followed by a comprehensive analysis that included PCA, conformational changes, salt bridge, hydrogen bond, hydrophobic, residue pair correlation network, and electrostatic potential analysis. We found monitoring the minimum distances of HBC and G loop gates, and K^+^ ion permeation reflects GIRK2 channel gating activity. All the three systems were started from the same initial cryo-EM structure of GIRK2, which was bound with DiC8 PIP_2_ molecules and CHS, stabilizing the channel structure in an open state. During the MD simulations, we observed the both HBC and G loop gates were closed in the GIRK2-APO state, and there was no K^+^ ion permeation (Figure 2A). In the presence of PIP_2_, we observed both gates were near open states (around 6 Å), and two K^+^ ion permeation events. Although, the minimum gate distances are smaller than the initial cryo-EM structure (Figure 2B). In the presence of PIP_2_ and CHS, we observed both gates were opened, and the minimum distances of the gates were near to the initial cryo-EM structure. 43 K^+^ ions were observed passing through both gates of GIRK2 channel (Figure 2C). The results indicate that CHS facilitates GIRK2 channel opening in the presence of PIP_2_.

In the GIRK2-APO state during the MD simulations, two K^+^ ions occupied the SF region for most of the time. In contrast, the GIRK2/PIP_2_ and GIRK2/PIP_2_/CHS systems showed a clear increase in the proportion of single K^+^ ion occupation. In the central cavity region, all three systems showed occupation by one, two, or three K^+^ ions. However, occupation by four K^+^ ions was observed only in the GIRK2/PIP_2_ and GIRK2/PIP_2_/CHS systems (Figure 3A). These results suggest K^+^ ion transfer from the SF to the central cavity region of the channel in presence of PIP_2_ or PIP_2_/CHS. Permeation of K^+^ ions in both GIRK2/PIP_2_ and GIRK2/PIP_2_/CHS systems revealed that they spent most of their time in the SF region (near residues T154, I155 and G156), and less time in the central cavity and the two gate regions. It is worth noting that ions spent more time near to the residues E152 and N184 than any other residues in the central cavity region of the channel (Figures 3B, C). These two residues were previously identified as critical for GIRK2 channel function (Wydeven et al., 2014; Li et al., 2020; Gazgalis et al., 2022; Li et al., 2024). Experimentally, the GIRK2:E152D mutation significantly potentiates channel currents (Yi et al., 2001).

The combined PCA results highlight significant conformational changes occurring between the TMD (transmembrane domain) and CTD (cytosolic domain), and within CTD regions when comparing the GIRK2-APO and GIRK2/PIP_2_/CHS systems during the simulations (Figure 4). Hinge motions were identified by DynDom analysis, indicating that residues G70 and N71, and G189-S196, form a hinge between the TMD and CTD regions of GIRK2. The residues with the largest phi and psi torsional angle changes were identified as being primarily located in the CTD regions of the channel (Figure 5). Interestingly, residues D81 and V87 are located in the S-HLX, near the CHS binding site B, while residues R73 and E74 are situated just before the S-HLX. Residues Q248 and G252 are located in the βD-βE loop. Residues V264, G265, F274, and L275 are located in the E-G-F loop. Residues P340, V341, D346, and E350 are located in the βL-βM region. These residues exhibit significant phi/psi torsional angle changes when comparing the GIRK2-APO and GIRK2/PIP_2_/CHS systems, which reflects PIP_2_/CHS induced conformational changes for GIRK2 activation (Figure 5B).

By comparing the differences of salt bridge networks in both GIRK2-APO and GIRK2/PIP_2_/CHS states, we identified key salt bridge pairs that could play a critical role in GIRK2 channel activation (Supplementary Figure S5). For example, E236 interacts with R272 and points to the inside of the tunnel in the GIRK2/PIP_2_/CHS state to facilitate K^+^ ion permeation, while it forms salt bridges with residues R324, R272, R240, and K242 in the GIRK2-APO system. E315 forms salt bridge interactions with K199 in the GIRK2-APO state but switches interactions to R324 in the GIRK2/PIP_2_/CHS state. K199 also forms salt bridges with D88 in S-HLX in the Apo state but not in the PIP_2_/CHS bound state. Releasing K199 to coordinate PIP_2_ is required for channel activation. Salt bridges formed between K247 (βE) and E302/E303 (10 residues upstream of the G-loop gate) may facilitate channel activation. Weakened salt bridge interactions between E150 (PH) and R160 (SF) in the GIRK2/PIP_2_/CHS system could facilitate K^+^ ion permeation in the SF region.

From hydrogen bond network analysis, we identified that critical HB pairs for GIRK2 activation by PIP_2_/CHS. For example, Y58 (N-ter) interacts with E305 (βH) in the Apo state but interacts with S277 (EGF) in the PIP_2_/CHS bound state through hydrogen bonds. H69 (βA)-D228 (CD) shows increased hydrogen bond formation in the Apo state, while H68 (βA)-N231 (CD) shows increased hydrogen bond formation in the PIP2/CHS bound state. Additionally, N66 (βA) increases hydrogen bond formation with Y349 (βM) in the PIP_2_/CHS state, indicating that the N-terminus could be involved in channel activation. Two hydrogen bond restrictions, Y78 (S-HLX)-L229 (CD) and V87 (S-HLX)-Q197 (B loop), are formed in the Apo state but weakened in the PIP_2_/CHS bound state. E152 (PH) interacts with W106 (TM1) through a hydrogen bond in the Apo state but is released to interact with K^+^ ions in the PIP_2_/CHS bound state. Two additional hydrogen bonds, R240 (βD)-L273 (EGF) and E315 (G loop)-H233 (CD), are formed in the PIP_2_/CHS bound state, which may facilitate G loop opening for channel activation (Supplementary Figure S6). The E315 (G loop)-H233 (CD-loop) interaction was previously identified during Na^+^-mediated effects on the channel gates (Li et al., 2019).

Through hydrophobic interaction analysis, we identified critical hydrophobic pairs for the channel activation. For example, the HBC residue, F192 forms hydrophobic interactions with F192 from adjacent subunits in the Apo state, but the interactions are disrupted in the PIP_2_/CHS bound state, which is consistent with the HBC gate opening for channel activation (Figure 1). Hydrophobic pairs, M187 (TM2)-A185 (TM2 of adjacent subunits), F186 (TM2)-F98 (TM1), I149(PH)-L173 (TM2), L172 (TM2)-I112 (TM1) and Y349 (βM)-P256 (βE, adjacent subunit) are formed in the Apo state, but are disrupted in the PIP_2_/CHS bound state for channel activation. There are clearly increased hydrophobic interactions between TM2 and the S-HLX in the PIP_2_/CHS bound state, including C190 (TM2)-F83(S-HLX), S196 (TM2)-F83(S-HLX), and F186 (TM2)-I82(S-HLX). Additionally, there are increased hydrophobic interactions between M187 (TM2)-V101 (TM1) and I195 (TM2)-W91 (TM1) in the PIP_2_/CHS bound state. It's worth noting that F98 (TM1) forms a hydrogen bond interaction with F186 (TM2) in the Apo state, but it switches to interact with hydrophobic residues M191 (TM2) and V178 (TM2) in the PIP_2_/CHS bound state. In addition, hydrophobic pairs I309 (βH)-F274 (EGF), F274-K242 (βD), and Y353 (βM)-V206(B loop) in the CTD are formed in the PIP_2_/CHS bound system. These hydrophobic interactions stabilize the channel in its open state.

By comparing residue pair correlations based on the MD simulations, we identified changes in correlated residue pairs upon PIP_2_/CHS binding. A few correlated regions can be highlighted: S-HLX residues (near the PIP_2_ binding site) show increased positive correlations with SF, TM1, TM2, and HBC of the transmembrane region, as well as with βM, βG, and EGF regions of the CTD. The results may indicate the central regulatory role of S-HLX in channel activation, enabled by PIP_2_ to coordinate both the HBC and G-loop gates as well as the SF for channel opening and K^+^ ion permeation. G loop residues show increased positive correlations with residues on TM1 and the linker residue between TM2 and the CTD. It also shows increased negative correlations upon PIP_2_/CHS binding. For example, negative correlations are observed between N-terminal residues and βB, βH, and βN regions; TM1 and PH/TM2; TM2 and SF; and βN and βD/βE regions.

Two CHS binding sites were identified by Cryo-EM studies. During the MD simulations, the CHS molecules in these two sites remain stable and interact with the binding site residues. In site A, the CHS mainly interacts with residues in TM1 and TM2. In site B, the CHS interacts with residues in S-HLX, TM1, TM2, and the CD loop (Figure 14). It appears that both CHS binding sites are required to achieve the maximum effect of channel activation. We conducted two additional independent MD simulations with single binding sites bound with CHS/PIP_2_. However, only 4 and 3 K^+^ ion permeation events were observed for the GIRK2/PIP_2_/CHS-siteA and GIRK2/PIP_2_/CHS-siteB systems, respectively (Supplementary Figure S11). The percentages of HBC gate openings for GIRK2-APO, GIRK2-PIP_2_, GIRK2/PIP_2_/CHS-siteA, GIRK2/PIP_2_/CHS-siteB, and GIRK2/PIP_2_/CHS-siteAB are 0.1%, 34.0%, 88.9%, 87.0%, and 91%, respectively. The percentages of G loop gate openings for GIRK2-APO, GIRK2-PIP_2_, GIRK2/PIP_2_/CHS-siteA, GIRK2/PIP_2_/CHS-siteB, and GIRK2/PIP_2_/CHS-siteAB are 71.8%, 65.2%, 79.0%, 91.2%, and 82.9%, respectively [based on a gate distance cutoff of 5.69 Å, (Li et al., 2019)]. The results indicate that CHS binding to both sites facilitates HBC gate opening more effectively compared to CHS binding at a single site. CHS binding at site B facilitates G loop gate opening more effectively compared to binding at site A. It is worth mentioning that M187 (TM2, the corresponding residue is M182 in GIRK4) is located in the CHS binding site B. Our previous studies showed that M182 in GIRK4 was identified as a key molecular switch for cholesterol potentiation. Mutating M182 in GIRK4 to the corresponding residue Ile in Kir2.1 converted the effect of cholesterol from potentiation to suppression (Corradi et al., 2022).

Our MM-GBSA-based binding free energy calculation results show that the binding of CHS enhances the PIP_2_ and channel interactions, which is consistent with previously published experimental results (Glaaser and Slesinger, 2017). Compared with the GIRK2/PIP_2_ system, PIP_2_ increases interactions with binding site residues K194, R92, K90, and R60, while decreasing interactions with residues K64, K200, R324, and R338 in the GIRK2/PIP_2_/CHS system (Figure 15). Similar results had been observed in our previous studies, for example, Gβγ enhances K194 but decreases K200 and PIP_2_ interactions (Li et al., 2019). Increasing PIP_2_ and channel interactions facilitates GIRK2 channel opening and activation.

To better understand PIP_2_ and channel interactions in regulating channel activation, we calculated the internal electric field in the GIRK2 channel in the absence and presence of PIP_2_. By comparing the electrostatic potential of the channel, we found that the highly negatively charged PIP_2_ molecules (-5e each) alter the internal electric field and lower the negative free energy barrier for K^+^ ions, thereby facilitating K^+^ ion permeation (Figure 16).

## Conclusion

We conducted 1µs MD simulations on GIRK2-APO, GIRK2/PIP_2_ and GIRK2/PIP_2_/CHS systems to understand GIRK2 channel activation regulated by CHS in the presence of PIP_2_. We found that CHS binding facilitates GIRK2 channel opening, with 43 K^+^ ion permeation events observed, compared to 0 and 2 K^+^ ion permeation events for GIRK2-APO and GIRK2/PIP_2_, respectively. We performed a comprehensive analysis of MD simulation results, including PCA, conformation changes, salt bridge, hydrogen bond, hydrophobic residue pair correlation network, binding sites of CHS and PIP_2_, and electrostatics potential of the channel. Residue pairs forming salt bridges, hydrogen bonds, hydrophobic interactions, and correlations, which are critical for GIRK2 channel activation upon PIP_2_/CHS binding, were identified through interaction network analysis. MM-GBSA binding energy calculations show that the binding of CHS to the GIRK2 channel enhances PIP_2_ interactions with the channel, consistent with previous experimental results. The negatively charged PIP_2_ also alters the internal electrostatic potential field in the channel and lowers the negative free energy barrier for K^+^ ion permeation.

## Data Availability

The raw data supporting the conclusions of this article will be made available by the authors, without undue reservation.

## Abbreviations

GMD, gating molecular dynamics; PCA, Principal Component Analysis; cryo-EM, cryo-electron microscopy; RMSF, root mean square fluctuation; RMSD, root mean square deviation; CHS, cholesteryl hemisuccinate; PIP_2_, Phosphatidylinositol 4,5-bisphosphate; HBC, helix bundle crossing; EV, eigenvector; HP, hydrophobic; SB, salt bridge; HB, Hydrogen bond; SF, selectivity filter; IFD, induced-fit-docking; PTX, pertussis; AF, atrial fibrillation; LQT, Long QT; VTA, ventral tegmental area; TMD, transmembrane domain; CTD, cytosolic domain.
