# Supplementary material for: Molecular mechanism of GIRK2 channel gating modulated by cholesteryl hemisuccinate
Source: Front Physiol. 2024 Oct 18;15:1486362. doi: 10.3389/fphys.2024.1486362 (PMC11527606; doi:10.3389/fphys.2024.1486362)
Supplement: Supplementary file 1 [file DataSheet1.pdf]

Supplementary Material

Molecular mechanism of GIRK2 channel gating modulated by cholesteryl hemisuccinate

Meng Cui[1,2]*, Yongcheng Lu[1], Xinyi Ma[1], Diomedes E. Logothetis[1,2,3,4,5]*

[1]Department of Pharmaceutical Sciences, School of Pharmacy, Bouvé College of Health Sciences, Northeastern University, Boston, Massachusetts, 02115, USA

[2]Center for Drug Discovery, Northeastern University, Boston, MA 02115, USA

[3]Affiliate of Chemistry and Chemical Biology, Northeastern University, Boston, MA 02115, USA

[4]Affiliate of Bioengineering, Northeastern University, Boston, MA 02115, USA

[5]Affiliate of Roux Institute of Northeastern University, Portland, ME 04101, USA

* To whom correspondence should be addressed: m.cui@northeastern.edu (MC); d.logothetis@northeastern.edu (DEL)

```
1    MTMAKLTESMTNVLEGDSMDQDVESPVAIHQPKLPKQARDDLPRHISRDRTKRKIQRYVR  60

           -βA-          --S-HLX---      -----------TM1-----------
61   KDGKCNVHHGNVRETYRYLTDIFTTLVDLKWRFNLLIFVMVYTVTWLFFGMIWWLIAYIR  120

   3/10h                    --Pore HLX----SF----      -----TM2-----
121  GDMDHIEDPSWTPCVTNLNGFVSAFLFSIETETTIGYGYRVITDKCPEGIILLLIQSVLG  180

     ----------HBC---B-loop-       --βB--  ---βC---CD loop--βD---
181  SIVNAFMVGCMFVKISQPKKRAETLVFSTHAVISMRDGKLCLMFRVGDLRNSHIVEASIR  240

     --------     ---βE-------E-G-F loop-----βG--           -αE--
241  AKLIKSKQTSEGEFIPLNQSDINVGYYTGDDRLFLVSPLIISHEINQQSPFWEISKAQLP  300

     -   -βH-----G loop---     3/10h-βJ βK-   -βL-   -βM-      βN
301  KEELEIVVILEGIVEATGMTCQARSSYITSEILWGYRFTPVLTMEDGFYEVDYNSFHETY  360

     --      ----αF--------
361  ETSTPSLSAKELAELANRAEVPLSWSVSSKLNQHAELETEEEEKNPEELTERNGDVANLE  400


420  NESKV                                                         425
```

Figure S1. Sequence of mouse GIRK2 channel (UniProtKB accession: P48542) marked with secondary structure regions. TM: transmembrane helix; HLX: helix; SF: selectivity filter; HBC: helix bundle crossing.

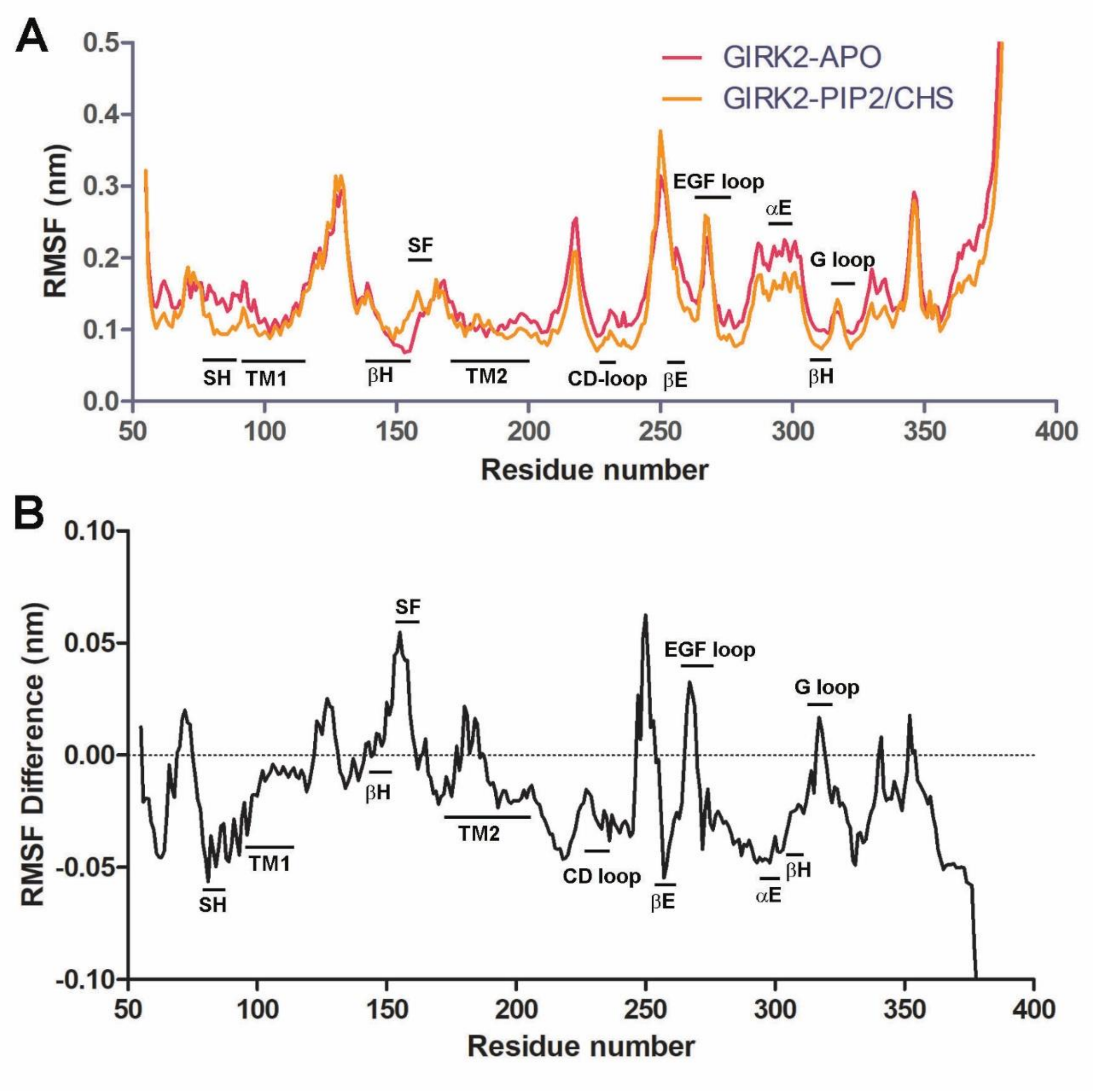

Figure S2. Root mean square fluctuation (RMSF) of the Cα atoms of GIRK2-APO, and GIRK2-PIP_2-CHS based on the MD simulations (200 – 1000 ns). **A.** Average RMSF for GIRK2-APO and GIRK2-PIP_2-CHS. **B.** RMSF difference between GIRK2-APO and GIKR2-PIP_2-CHS.

Table S1. Dwell time of K^+ ions in the GIRK2 channel.

	0 ion	1 ion	2 ions	3 ions	4 ions
**GIRK2-APO (SF)**		0.64	99.33	0.04	
**GIRK2-PIP2 (SF)**		29.40	70.60		
**GIRK2-PIP2-CHS(SF)**		32.96	67.0	0.04	
**GIRK2-APO(SF-HBC)**		16.16	42.89	40.94	
**GIRK2-PIP2 (SF-HBC)**		15.00	15.97	47.58	21.45
**GIRK2-PIP2-CHS(SF-HBC)**	0.30	14.89	51.56	31.61	1.65

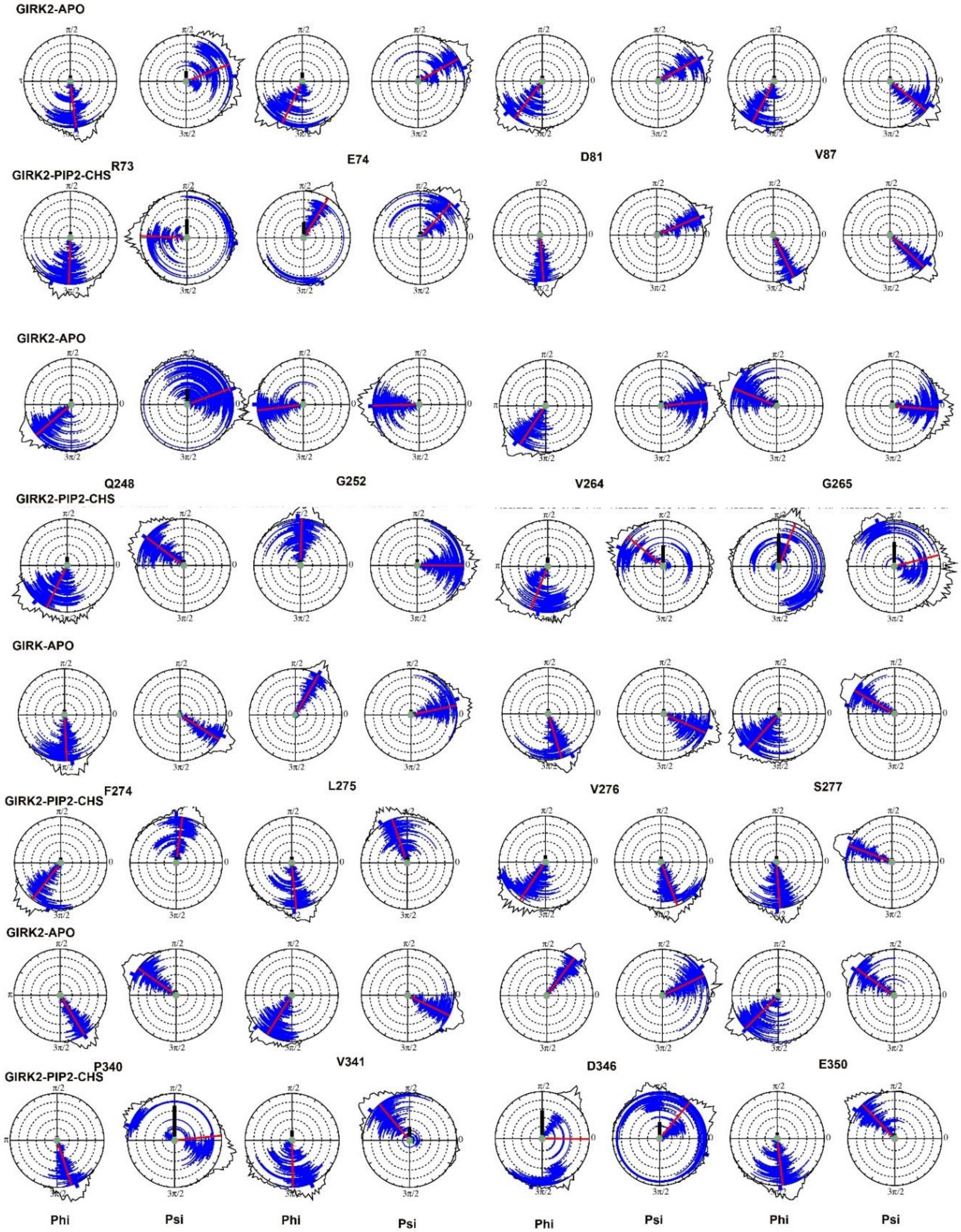

Figure S3. Dial plots for phi/psi angle distributions of the selected residues for GIRK2/APO, GIRK2/PIP_2/CHS based on MD simulations (200 – 1000ns). The center of the plot represents the starting point of the simulation. The blue line depicts the trajectory (starting at the center and ending at the outer circle), while the red line indicates the average of the measured angle.

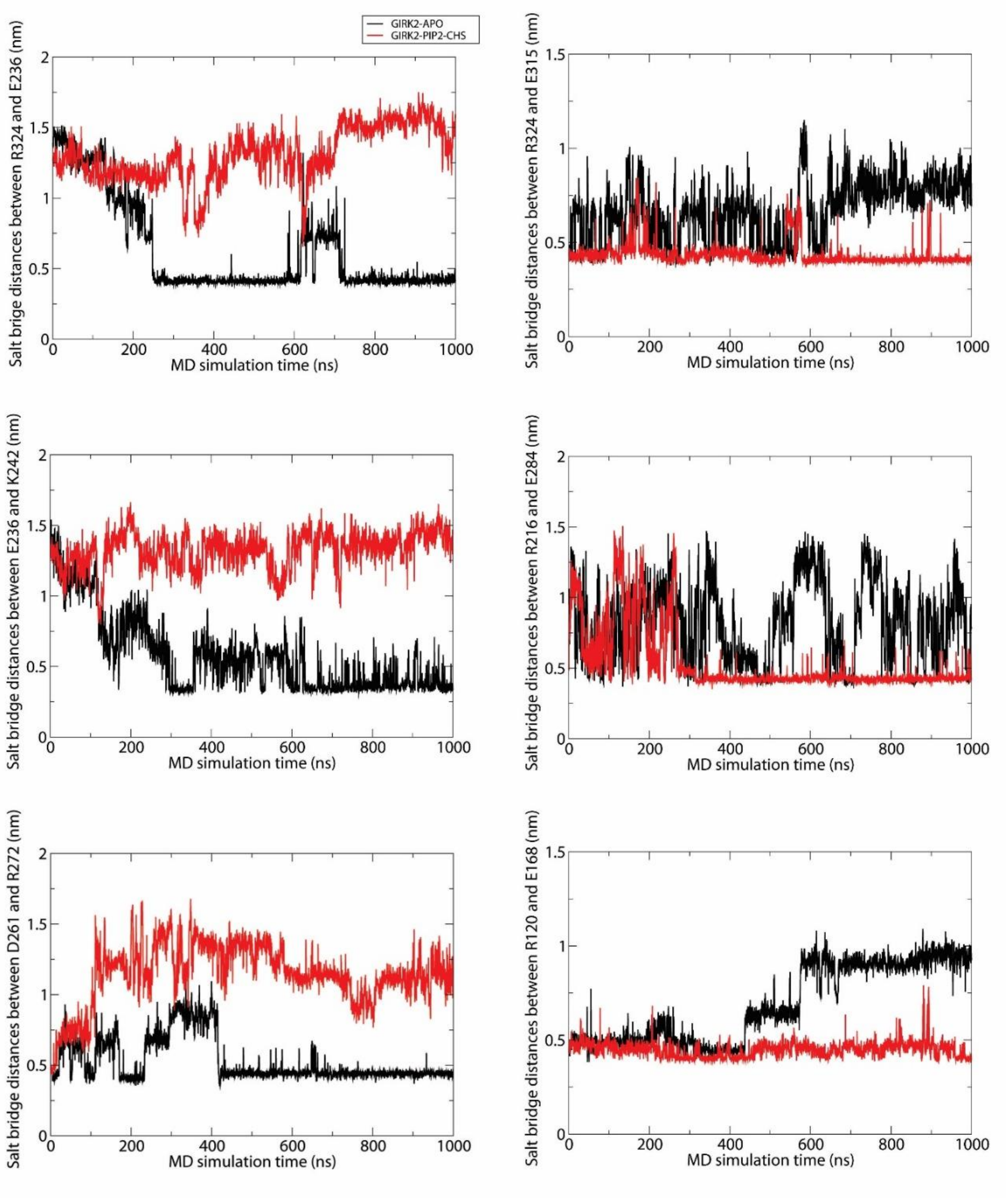

Figure S4. Selected key different salt bridge residues in GIRK-APO, and GIRK2-PIP_2-CHS. Plots of distances as function of time during 1μs MD simulations.

Table S2. Selected key different salt bridge residues in GIRK-APO, and GIRK2-PIP2-CHS (dif >0: SB formed in APO, broken in PIP_2/CHS; dif<0: SB broken in APO, formed in PIP_2/CHS).

Res1	Res2	dif	Res1	Res2	dif	Res1	Res2	dif
R324•B	E236•C	0.86	K245•B	E302•B	0.49	K247•B	E302•B	-0.47
K199•B	E315•C	0.84	E236•B	R272•B	-0.39	D217•A	K247•D	-0.52
D261•C	R272•D	0.79	K247•C	E303•C	-0.39	R120•D	E168•D	-0.54
D88•A	K199•A	0.78	D164•C	R160•D	-0.39	R337•A	E253•D	-0.56
E236•A	R240•D	0.75	K61•D	E331•D	-0.39	R216•A	E284•A	-0.58
E236•A	K242•D	0.73	K247•C	E302•C	-0.41	K245•D	E302•D	-0.58
R57•B	E305•B	0.66	D81•B	R201•C	-0.41	R324•C	E315•D	-0.58
R120•C	E168•C	0.64	K242•A	D270•B	-0.42			
R216•C	E284•C	0.62	E150•D	R160•D	-0.42			
R216•A	E251•D	0.6	D270•A	K242•D	-0.42			
E315•A	K199•D	0.59	E236•C	R272•C	-0.45			
E253•A	R216•B	0.56	E150•C	R160•C	-0.46			
K199•A	E315•B	0.51	R324•B	E315•C	-0.46			
R201•A	D81•D	0.5	R272•A	D271•D	-0.46			

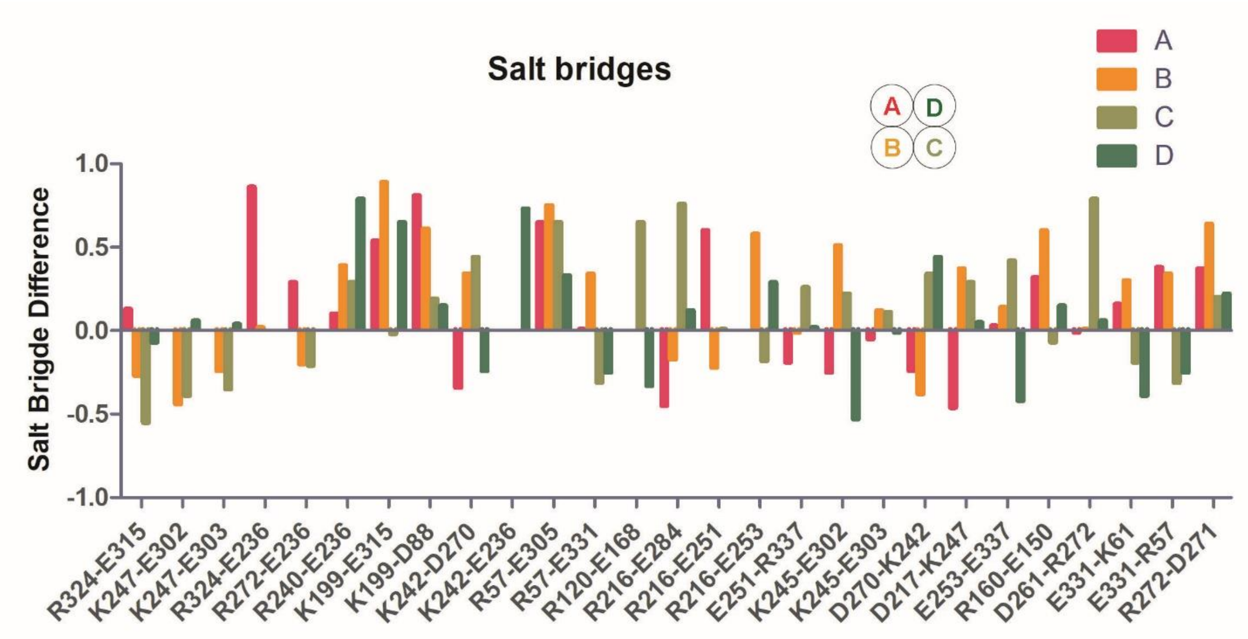

Figure S5. Salt bridge difference between GIRK-APO, and GIRK2-PIP_2-CHS systems. (dif >0: SB formed in APO, broken in PIP_2/CHS; dif<0: SB broken in APO, formed in PIP_2/CHS).

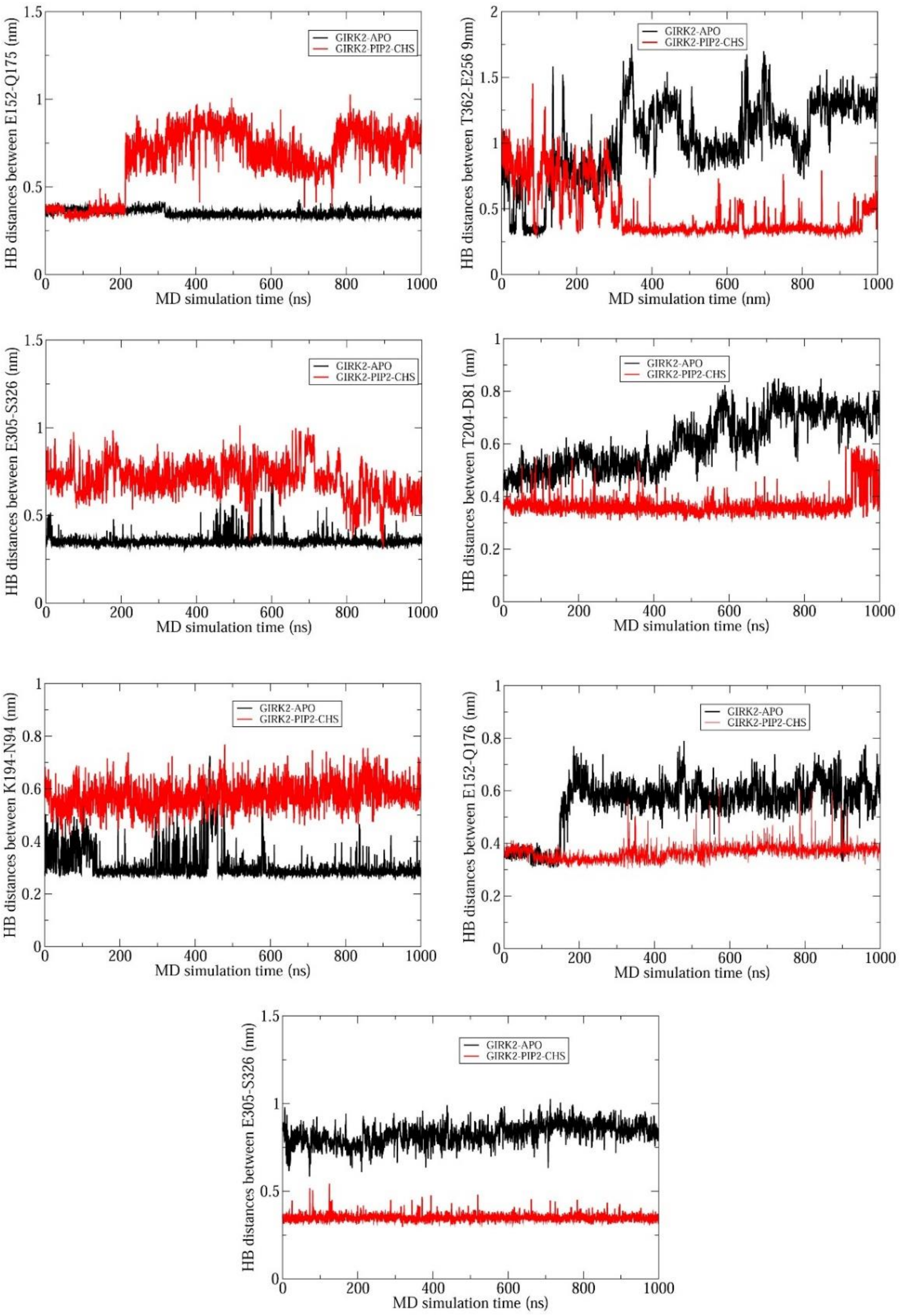

Figure S6. Selected key different hydrogen bond residues in GIRK-APO, and GIRK2-PIP_2-CHS. Plots of distances as function of time during 1μs MD simulations.

Table S3. Selected key different hydrogen bond residues in GIRK-APO, and GIRK2-PIP$_2$-CHS (dif >0: HB formed in APO, broken in PIP$_2$/CHS; dif<0: HB broken in APO, formed in PIP$_2$/CHS).

Res1	Res2	dif	Res1	Res2	dif	Res1	Res2	dif
I255•B	K247•B	1	E305•B	Y58•B	0.84	R240•D	L273•A	-0.86
Q176•D	E152•D	0.99	T151•D	Y157•A	0.84	L273•B	R240•A	-0.87
Y78•D	L229•A	0.97	G156•D	E150•D	0.84	G169•C	P167•C	-0.87
S326•B	E305•B	0.94	D270•B	T268•B	0.83	E253•A	T249•A	-0.88
E253•C	T249•C	0.94	T80•C	Y76•C	0.83	Q259•D	F274•A	-0.88
E253•B	T249•B	0.93	E315•C	K199•B	0.83	T204•C	D81•B	-0.9
N94•B	L86•B	0.92	T80•D	E203•A	-0.8	E311•D	E236•A	-0.91
I171•C	P167•C	0.92	I171•C	E168•C	-0.81	L86•A	I82•A	-0.92
G189•D	A185•D	0.92	V178•D	L174•D	-0.81	Q176•C	E152•C	-0.96
G189•A	A185•A	0.91	Y78•B	V72•B	-0.83	T85•A	D81•A	-0.99
P256•C	Y58•C	0.91	N258•D	K245•D	-0.83	T80•B	Y76•B	-0.99
I255•C	K247•C	0.89	T204•B	D81•A	-0.84	S326•C	E305•C	-0.99
E152•B	W106•B	0.87	Y349•D	N66•C	-0.84			
V72•B	G70•B	0.85	E331•D	A211•D	-0.85			

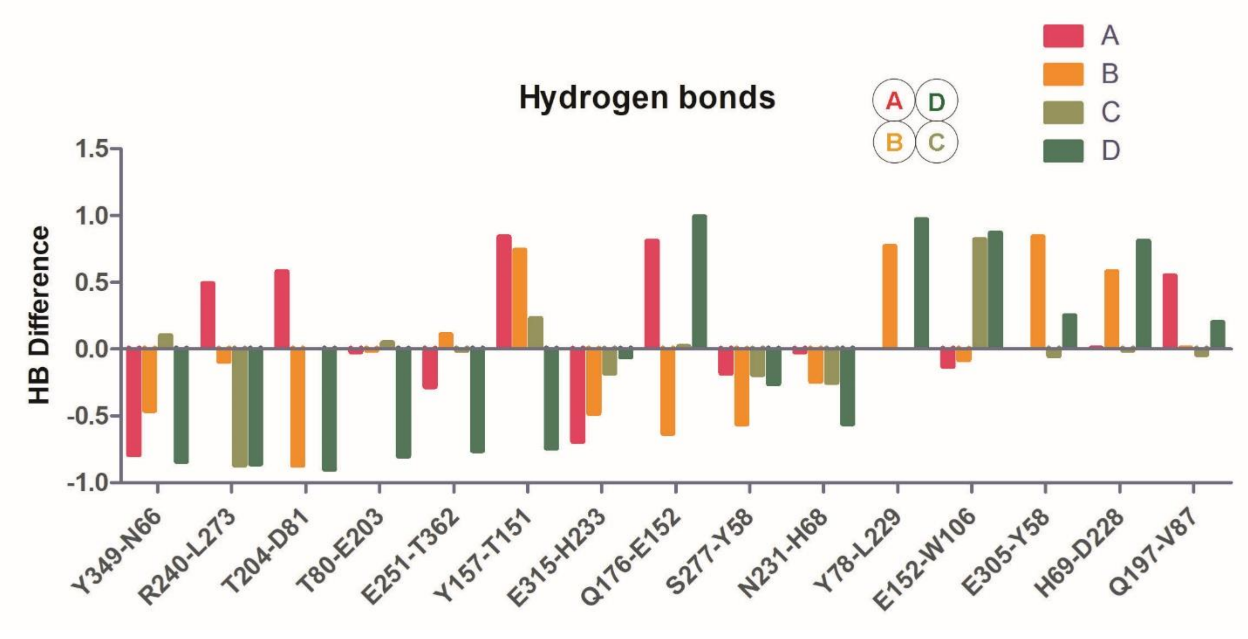

Figure S7. Hydrogen bond difference between GIRK-APO, and GIRK2-PIP2-CHS systems. (dif >0: HB formed in APO, broken in PIP$_2$/CHS; dif<0: HB broken in APO, formed in PIP$_2$/CHS).

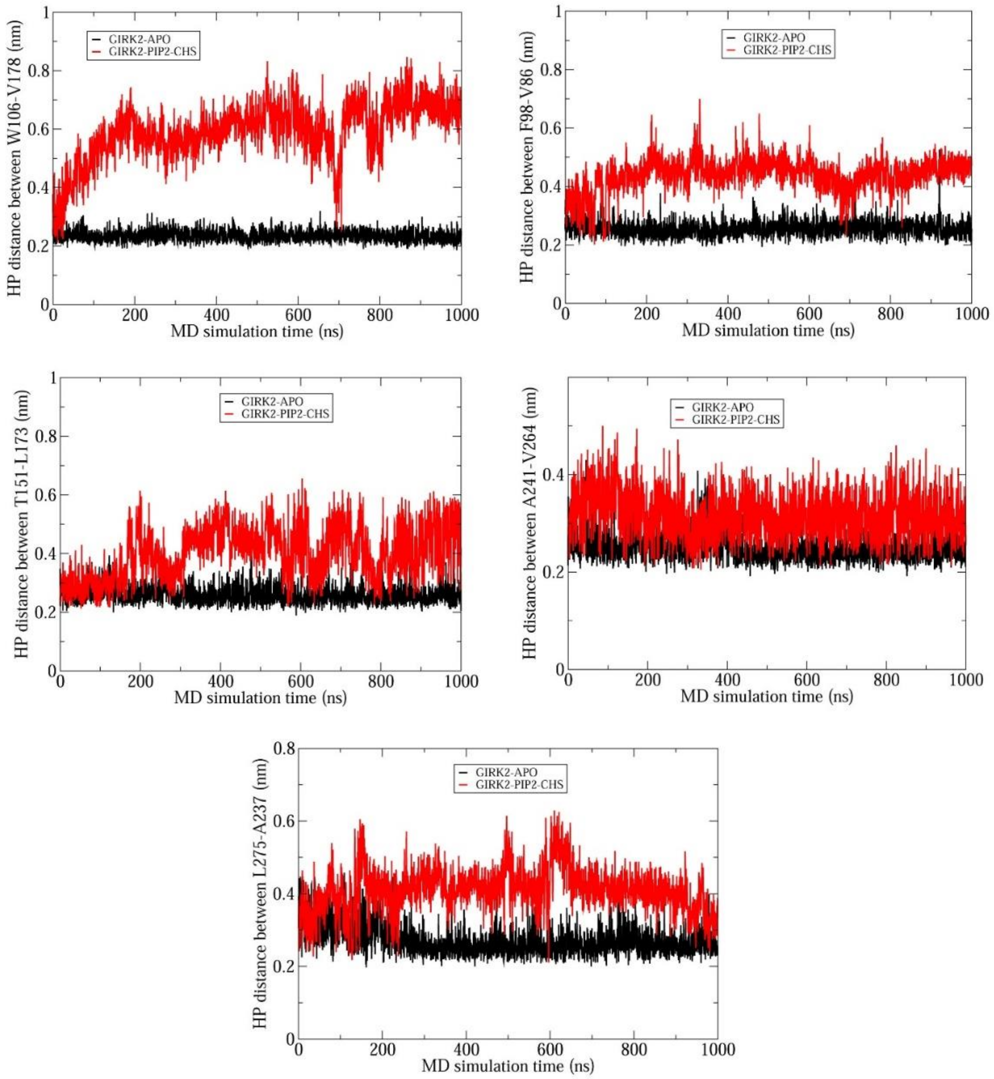

Figure S8A. Selected key different hydrophobic residues in GIRK-APO, and GIRK2-PIP$_2$-CHS. Plots of distances as function of time during 1μs MD simulations.

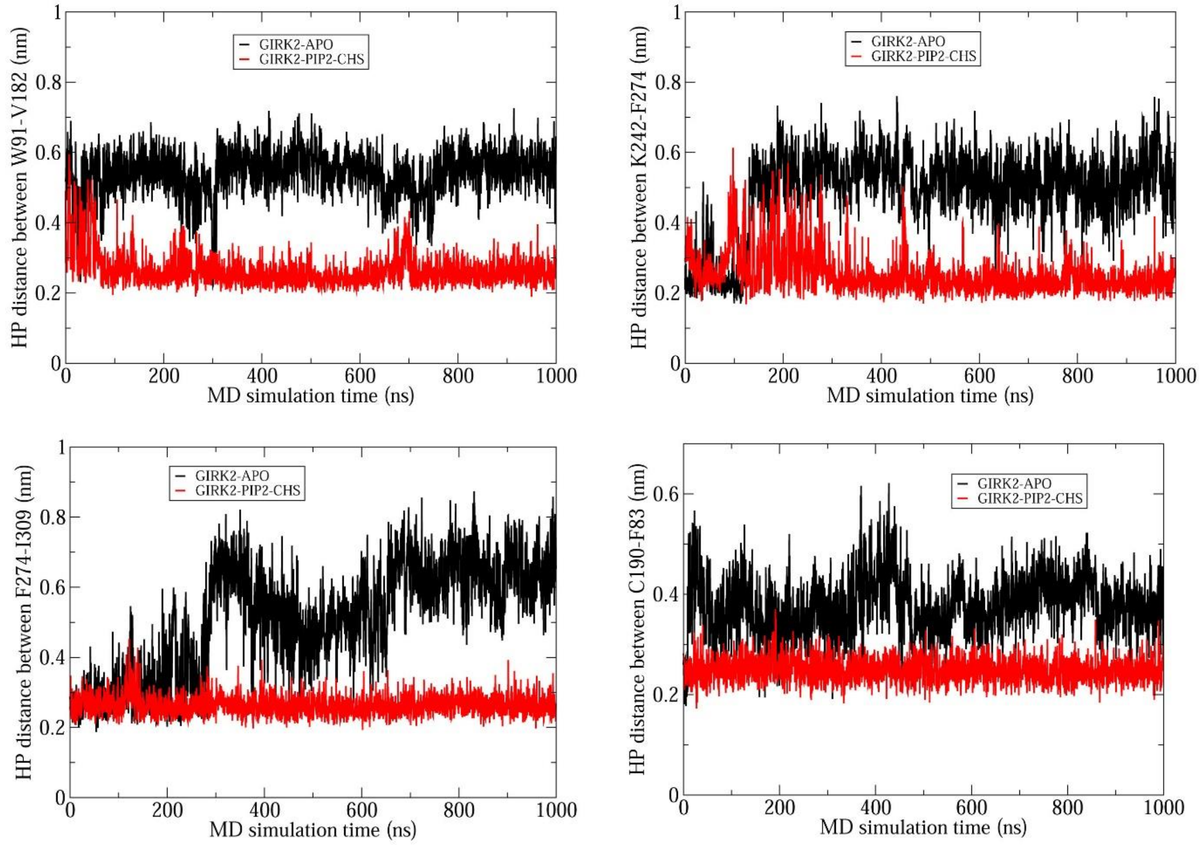

Figure S8B. Selected key different hydrophobic residues in GIRK-APO, and GIRK2-PIP_2-CHS. Plots of distances as function of time during 1μs MD simulations.

Table S4. Selected key different hydrophobic residues in GIRK-APO, and GIRK2-PIP$_2$-CHS (dif >0: HP formed in APO, broken in PIP$_2$/CHS; dif<0: HP broken in APO, formed in PIP$_2$/CHS).

Res1	Res2	dif	Res1	Res2	dif	Res1	Res2	dif
R230•C	Y78•B	0.87	I255•B	T249•B	0.66	M191•A	F98•A	-0.64
F192•B	F192•A	0.86	L275•A	A237•A	0.65	L229•C	V72•B	-0.67
W106•B	V178•A	0.85	I149•B	L174•A	0.64	I195•B	W91•B	-0.67
F192•C	F192•B	0.79	F192•D	F192•A	0.64	M187•A	V101•A	-0.68
F98•A	L86•A	0.74	F348•B	L257•A	0.63	Q322•D	V235•A	-0.69
L173•C	T151•C	0.74	Y157•D	E150•D	0.63	M191•B	F98•B	-0.72
F145•B	L174•A	0.73	M191•B	A185•A	-0.58	F274•C	K242•B	-0.73
Y102•D	L174•C	0.71	F83•D	S196•A	-0.58	F192•B	G189•A	-0.74
F192•D	F192•C	0.71	Y266•D	R272•A	-0.58	V307•D	F274•A	-0.79
V264•D	A241•D	0.69	V193•D	T317•A	-0.59	I309•D	F274•A	-0.85
G169•C	W113•C	0.69	L146•D	L173•C	-0.59	C190•A	F83•A	-0.9
M187•B	A185•A	0.67	L257•D	I244•D	-0.59	C190•D	F83•D	-0.91
V188•B	V188•A	0.67	V235•B	E311•A	-0.61			
F186•D	F98•D	0.66	W91•B	I182•A	-0.63			

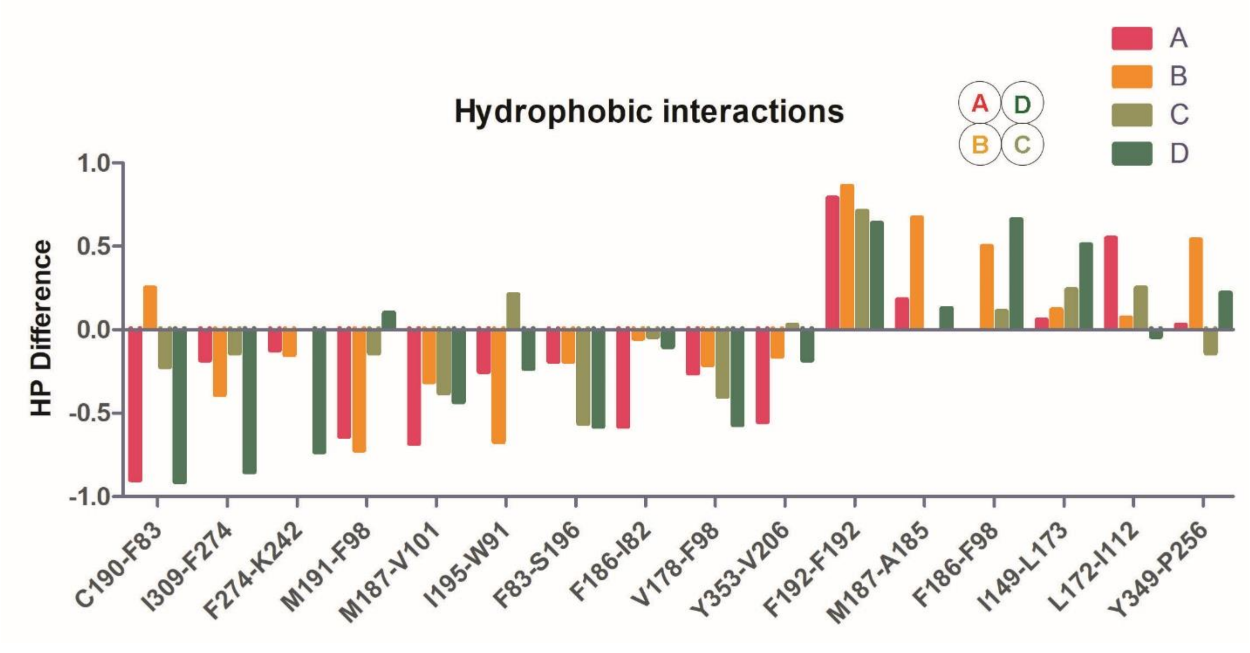

Figure S9. Hydrophobic interaction difference between GIRK-APO, and GIRK2-PIP2-CHS systems. (dif >0: HP formed in APO, broken in PIP$_2$/CHS; dif<0: HP broken in APO, formed in PIP$_2$/CHS).

Table S5. Selected key different residue correlations in GIRK-APO, and GIRK2-PIP_2-CHS (dif >0: positive correlations in APO, negative correlations in PIP_2/CHS; dif<0: negative correlations in APO, positive correlations in PIP_2/CHS).

Res1	Res2	dif	Res1	Res2	dif	Res1	Res2	dif
S325•B	G63•B	-1.0063	T153•B	I171•A	-0.929	L205•B	I82•A	0.7868
L146•B	S177•A	-1.0049	K64•B	V59•B	-0.9282	T204•B	I82•A	0.7931
L146•B	L174•A	-0.9778	S143•B	S177•A	-0.9242	L229•B	I82•A	0.7947
S326•B	G63•B	-0.9724	E150•B	L174•A	-0.9232	N354•A	F348•A	0.8134
T153•B	I112•B	-0.9716	F145•B	S177•A	-0.9229	R230•B	I82•A	0.8279
I149•B	L174•A	-0.9627	T154•B	I112•B	-0.9126	G70•D	T343•A	0.8437
I149•B	S177•A	-0.9584	L257•D	K247•D	0.7643	F348•B	S148•A	0.7639
R324•B	G63•B	-0.9582	E74•D	Y349•A	0.7686			
T153•B	L115•B	-0.9505	R73•D	F348•A	0.7735			
T153•B	I119•B	-0.9479	R230•B	F83•A	0.7735			
V142•B	S177•A	-0.9447	V72•D	Y349•A	0.7763			
T153•B	M111•B	-0.9427	I262•A	K90•A	0.7847			
T209•B	G63•B	-0.9407	N71•D	V264•A	0.7849			
Y327•B	G63•B	-0.9348	Y78•D	T268•A	0.7858			

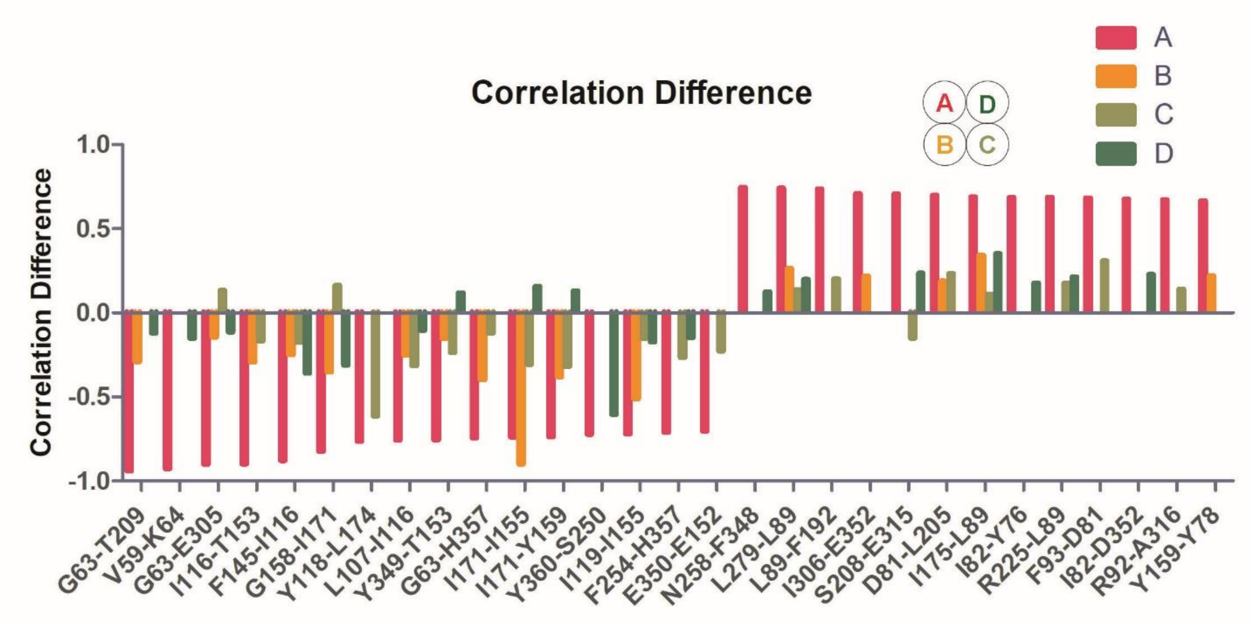

Figure S10. Correlation difference between GIRK-APO, and GIRK2-PIP_2-CHS systems. (dif >0: positive; dif<0: negative correlation formed in PIP_2/CHS).

Table S6: GIRK2/PIP2 MMGBSA interaction energies with PIP_2 (-80.70 kcal/mol)

Residue	Interaction Energy (Kcal/mol)	Residue	Interaction Energy (kcal/mol)
ARG388	-21.30	ILE182	-0.88
LYS199	-19.89	VAL99	-0.55
LYS64	-18.75	PHE108	-0.49
ARG324	-16.19	LYS194	-0.34
LYS200	-16.05	LEU96	-0.33
TRP91	-3.38	VAL178	-0.32
LYS90	-2.22	ARG92	-0.32
LEU95	-1.65	ILE112	-0.30
LEU179	-1.44	PHE109	-0.30
ILE175	-0.92		

Table S7: GIRK2/PIP2/CHS MMGBSA interaction energies with PIP_2 (-98.97kcal/mol)

Residue	Interaction Energy (Kcal/mol)	Residue	Interaction Energy (Kcal/mol)
LYS194	-25.60	LEU95	-0.86
LYS199	-21.31	ARG324	-0.73
LYS90	-16.18	PHE112	-0.73
LYS64	-12.64	LEU172	-0.68
ARG60	-9.80	PHE109	-0.66
LYS200	-9.62	ILE182	-0.49
ARG92	-7.21	LEU79	-0.48
TRP91	-2.02	VAL183	-0.32
LEU179	-1.57	TRP113	-0.30
GLN197	-0.98	ILE82	-0.29
ILE112	-0.90	GLN176	-0.28
ILE175	-0.88	VAL87	-0.23

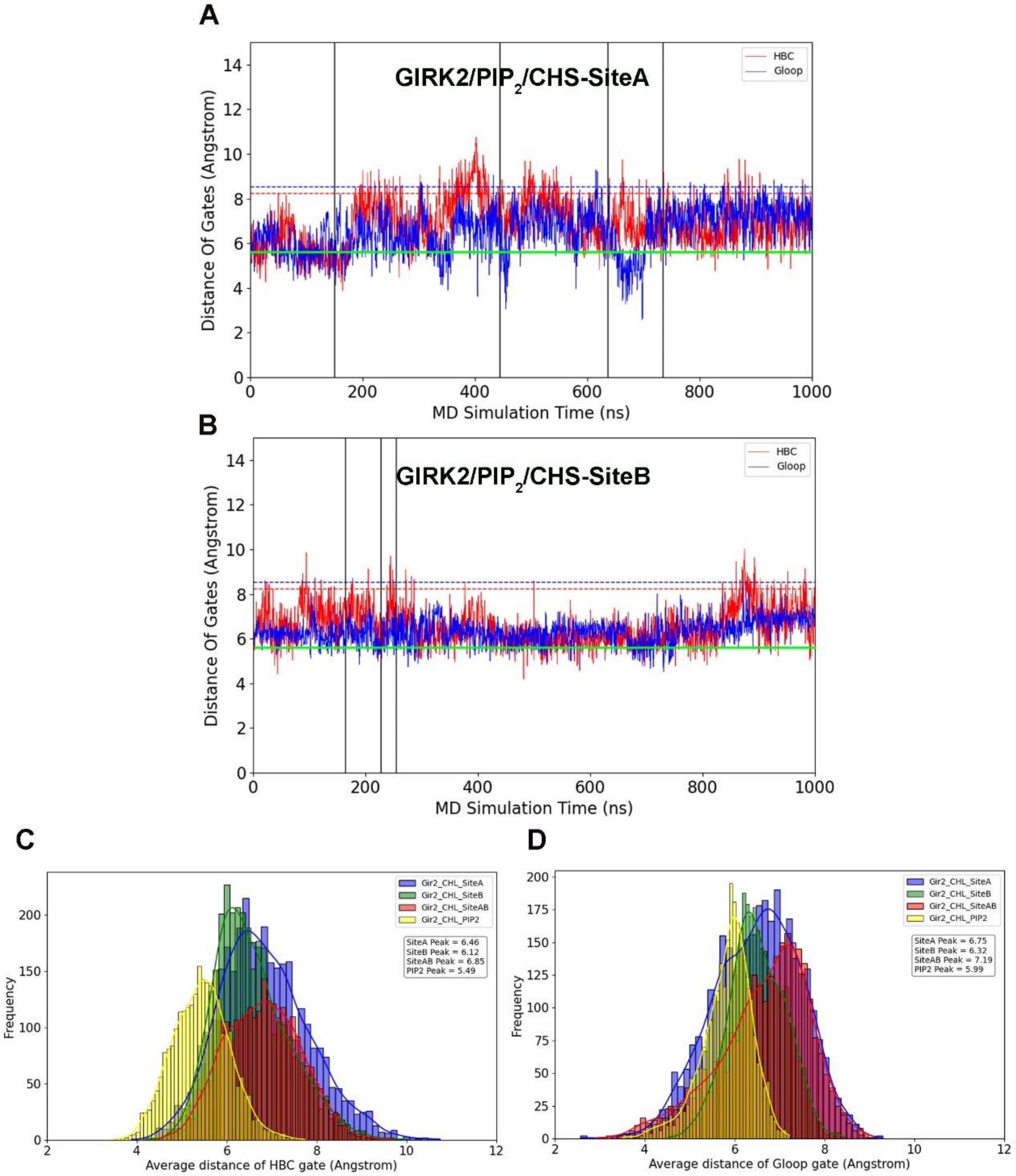

Figure S11. GIRK2 channel gating and K^+ ion permeation during MD simulations. **A.** GIRK2/PIP_2/CHS-SiteA. **B.** GIRK2/PIP_2/CHS-SiteB. Dish lines are gate distances of HBC (red) and G loop (blue) gates in the initial GIRK2 channel structure. The green lines are the cutoff distance (5.69 Å) for K^+ ion permeation as shown by Li et al., 2019). Vertical solid lines are the time points for K^+ ion permeation during the MD simulations (4 ions for GIRK2/PIP_2/CHS-SiteA, 3 ions for GIRK2/PIP_2/CHS-SiteB). **C.** Histogram of HBC gate; **D.** Histogram of G loop gate for GIRK2/PIP_2, GIRK2/PIP_2/CHS-SiteA, GIRK2/PIP_2/CHS-SiteB, and GIRK2/PIP_2/CHS-SiteAB systems.
